# Triaging of Culture Conditions for Enhanced Secondary Metabolite Diversity from Different Bacteria

**DOI:** 10.3390/biom11020193

**Published:** 2021-01-30

**Authors:** Jenny Schwarz, Georg Hubmann, Katrin Rosenthal, Stephan Lütz

**Affiliations:** Department of Biochemical and Chemical Engineering, TU Dortmund University, Emil-Figge-Straße 66, 44227 Dortmund, Germany; jenny.schwarz@tu-dortmund.de (J.S.); georg.hubmann@tudortmund.de (G.H.); katrin.rosenthal@tudortmund.de (K.R.)

**Keywords:** mass spectrometry, OSMAC approach, natural products, silent BGC activation, bioinformatics, screening

## Abstract

Over the past decade, the one strain many compounds (OSMAC) approach has been established for the activation of biosynthetic gene clusters (BGCs), which mainly encode the enzymes of secondary metabolite (SM) biosynthesis pathways. These BGCs were successfully activated by altering various culture conditions, such as aeration rate, temperature, and nutrient composition. Here, we determined the biosynthetic potential of 43 bacteria using the genome mining tool antiSMASH. Based on the number of BGCs, biological safety, availability of deposited cultures, and literature coverage, we selected five promising candidates: *Bacillus amyloliquefaciens* DSM7, *Corallococcus coralloides* DSM2259, *Pyxidicoccus fallax* HKI727, *Rhodococcus jostii* DSM44719, and *Streptomyces griseochromogenes* DSM40499. The bacteria were cultivated under a broad range of OSMAC conditions (nutrient-rich media, minimal media, nutrient-limited media, addition of organic solvents, addition of biotic additives, and type of culture vessel) to fully assess the biosynthetic potential. In particular, we investigated so far scarcely applied OSMAC conditions to enhance the diversity of SMs. We detected the four predicted compounds bacillibactin, desferrioxamine B, myxochelin A, and surfactin. In total, 590 novel mass features were detected in a broad range of investigated OSMAC conditions, which outnumber the predicted gene clusters for all investigated bacteria by far. Interestingly, we detected mass features of the bioactive compounds cyclo-(Tyr-Pro) and nocardamin in extracts of DSM7 and DSM2259. Both compounds were so far not reported for these strains, indicating that our broad OSMAC screening approach was successful. Remarkably, the infrequently applied OSMAC conditions in defined medium with and without nutrient limitation were demonstrated to be very effective for BGC activation and for SM discovery.

## 1. Introduction

Secondary metabolites (SMs) are a chemically diverse and large group of biomolecules with complex structures. Approximately 300,000 secondary metabolites are characterized and many SMs are biologically active [1]. They possess antibiotic, cytostatic, or other relevant activities [2,3]. Therefore, SMs are of high interest for drug development in pharmaceutical research. The genes that code for enzymes involved in microbial SM biosynthesis are often clustered in bacteria. The expression of these biosynthetic gene clusters (BGCs) is regulated by various parameters including growth phase and cell differentiation [1,4].

The traditional approach of natural product discovery from microorganisms comprises bioactivity-guided screenings of microbial extract libraries obtained from existing strain collections [5] or newly isolated microorganisms [6,7,8]. The members of the bacterial class actinomycetes are regarded as species with high biosynthetic potential of promising bioactive SMs [5]. However, the biosynthetic potential of an organism has been difficult to estimate owing to the lack of genomic information on BGCs. Thanks to the development of new sequencing technologies, the number of freely available fully sequenced genomes in public databases made genomic information more accessible for genome mining. To date, the National Center for Biotechnology Information (NCBI) provides more than 200,000 prokaryotic genome sequences with different depths of the genome assembly. In particular, the availability of genomes for organisms with high biosynthetic potential facilitated predictions with regards to potentially produced SMs. For example, the BGCs in genomes of interest can be analyzed using software tools like antiSMASH [9]. The antiSMASH tool exploits genomic information to mine previously unidentified natural products from the broad spectrum of the available sequenced genomes [10]. The genome mining approach enables a complete new route of SM discovery compared with the traditional approaches. Still a major challenge remains, which is the activation of the potential BGCs to enable the biosynthesis of the SMs by pathway enzymes present on the genome. BGC activation only occurs under certain cultivation conditions. Most often, the expression of BGCs is silenced in the organisms’ genomes [11]. One way to activate BGCs is through genetic modifications, e.g., ribosome engineering, the manipulation of regulators or quorum sensing systems, and heterologous expression [12,13]. Heterologous expression of BGCs has also been used to confirm these gene clusters as coding sequence of the natural biosynthesis pathway [14]. Aside from genetic modifications, BGC activation has been achieved by perturbing culture conditions, for example, co-cultivation [5]. The culture-condition induced approach is commonly known as the one strain, many compounds (OSMAC) approach to enable the discovery of many compounds produced by one microbial source [15].

The OSMAC approach comprises the alteration of easily adaptable culture conditions, like media composition, pH, temperature, the addition of enzyme inhibitors, oxygen supply, or culture vessel, to induce SM production during cultivation [15]. Table 1 summarizes three examples of pharmaceutically active new compounds obtained from successful OSMAC experiments using easily applicable changes in culture conditions. These changes in culture condition comprise variations of the media and of the carbon source.

Firstly, the BGC activation in OSMAC screenings is often related to variations in the type or concentration of nutrients. In particular, changes in carbon, nitrogen, or phosphate sources successfully induced the microbial production of SMs [19]. For example, Machushynets et al. only detected the quinazolinones A and B with an elevated glycerol concentration, but not on other tested carbon sources such as mannitol, fructose, or glucose [17]. Bode et al. showed that the addition of oat grains to the culture medium increased the number and diversity of produced cladospirones [16].

Secondly, additives such as solvents, heavy metals, tensides, precursors, and other small molecule elicitors were also used to successfully induce secondary metabolite production in microorganisms. Chen et al. reported that organic solvents influence the production of antibiotics. For example, increases in the production of tetracenomycin and thiostrepton by *Streptomyces* strains were obtained when adding dimethyl sulfoxide (DMSO) and ethanol (EtOH) to the culture broth [20]. Still, the underlying mechanism of solvent-induced BGC activation is not yet fully understood and one only can speculate on the mode of action of intracellular responses to solvents. DMSO might act on the translational level, induce stress proteins, or possess pheromone-like activity. Ethanol likely induces heat shock proteins [21] and permeabilizes the cell membrane [22].

Thirdly, Weinberg identified three heavy metals, Mn^2+^, Fe^3+^, and Zn^2+^, as key trace metals in secondary metabolism of bacteria and fungi. These metal ions induced the synthesis of bacterial exotoxins and peptide antibiotics [23]. Paranagama et al. showed the influence of the addition of heavy metal ions and heat shock on fungal secondary metabolite production profiles. Particularly, the plant-associated fungus *Paraphaeosphaeria quadriseptata* produced more monocillin I when the culture medium was supplemented with 0.5 mM ZnSO_4_ [24].

Finally, co-cultivation was used to successfully induce SM production in OSMAC screenings. The term co-cultivation designates the growth of two or more microorganisms at once, which triggers interactions between the organisms [25]. Cell–cell contact or the presence of small molecules presumably induced the expression of silent BGCs and the production of SMs [11,23,26]. It was shown that co-cultivation of various marine bacteria with terrestrial bacteria enhances antibiotic production [27]. Hence, OSMAC screenings became a successful alternative to activate BGCs through variations in culture conditions instead.

Despite the success of established OSMAC approaches in SM discovery, the variations in cultivation conditions are far from being exhaustively tested. Furthermore, although OSMAC studies were conducted with increasing numbers for SM discovery in the past years, our literature search has shown that some cultivation conditions are used more frequently. In that respect, the most frequently used OSMAC conditions to activate silent BGCs in bacterial cultures were different nutrient-rich media, followed by the addition of inducer molecules (n-acetylglucosamine, sub-inhibitory concentrations of antibiotics, and organic acids), and the use of co-cultivation [28,29,30,31,32,33,34]. Fewer studies changed the pH of the growth medium, applied temperature shocks during the cultivation, or added solvents and biotic additives such as heat-killed cells. While several studies investigated nutritional regulators and their control on secondary metabolism, only few studies investigated the secreted metabolome after the use of starvation conditions, referred to in the following as nutrient-limited OSMAC experiments [35,36]. Hence, these infrequently applied OSMAC conditions were not exhaustively explored for their potential to activate silent BGCs in organisms with a high biosynthetic potential or even well investigated organisms of the actinomycetes bacterial family.

In this study, we aimed to explore the less frequently used OSMAC conditions, like the nutrient-limited media experiments. To compare their potential for BGC activation and SM production to the more frequently applied OSMAC conditions, we analyzed the number and diversity of new mass features in high-performance liquid chromatography-mass spectrometry (HPLC-MS) analysis of extracts isolated from selected bacteria, which were grown under the various growth conditions. In total, five fully sequenced, but not thoroughly investigated bacteria were identified via genome mining, all of them sharing a high biosynthetic potential. The activation of their predicted BGCs was investigated using a broad range of culture conditions, including the less frequently applied OSMAC conditions. We used the well-established media variations as a benchmark of SM production in microbial cultures. Next, it was possible to evaluate the less frequently used culture conditions, i.e., Fe^3+^-, Mg^2+^-, and PO_4_^3−^-limitation, as well as addition of biotic additives and organic solvents, for their suitability to further increase the number of new mass features in bacterial cultures. Based on their potential to increase the number of new mass features and diversify the mass feature identity, the OSMAC conditions under PO_4_^3−^-limitation, Fe^3+^-limitation, defined M9 minimal medium, glucose mineral salt (GMS) medium, and several biotic additives were identified as conditions with a high potential to activate BGCs and SM production. Hence, these OSMAC conditions are promising starting points for broad screenings of other bacteria in the future. Furthermore, we noticed that 70% of the 590 new mass features were condition-dependent, i.e., they were only detected under exactly one culture condition. This observation led us to the conclusion that especially broad screenings of carefully selected strains are most promising for future SM discovery.

## 2. Materials and Methods 

### 2.1. Genome Mining 

The online-tool antiSMASH bacterial version 3.0 (Novo Nordisk Foundation Center for Biosustainability, Technical University of Denmark, Lyngby, Denmark) [9] was used to examine fully sequenced genomes of 42 bacteria. The percentage of the genomes dedicated to secondary metabolite production, classes of the encoded secondary metabolites, and the origin of the database BGCs were examined. In the Supplementary Material, all examined bacterial strains including their NCBI accession numbers are summarized in Table S1.

### 2.2. Organisms, Media, and Growth Conditions 

As reference or control group, all strains (*Bacillus amyloliquefaciens* DSM7, *Corallococcus coralloides* DSM2259, *Pyxidicoccus fallax* HKI727, *Rhodococcus jostii* DSM44719, and *Streptomyces griseochromogenes* DSM40499) were cultivated according to recommendations by DSMZ (German Collection of Microorganisms and Cell Cultures, Braunschweig, Germany). *B. amyloliquefaciens* was cultivated on nutrient broth (NB) medium [37], *C. coralloides* was grown on SP medium [38], and *P. fallax* on MD1 medium [39]. *R. jostii* was cultivated on tryptic soy broth (TSB) medium [40] and cultures of *S. griseochromogenes* were grown on glucose-, yeast-, malt- (GYM) medium [41] (Tables S3–S7). A detailed list of used chemicals and suppliers used to prepare the various growth media is presented in Table S2.

Cryo-cultures of all strains were stored at −20 °C in 10% (*v*/*v*) glycerol. Inoculum cultures of the bacteria were prepared by incubating 1 mL of the cryo-culture in 19 mL of medium in a baffled 100 mL flask on an orbital shaker at 150 rpm and 30 °C (28 °C for *R. jostii*) for 15–72 h depending on the bacterium. Different final biomasses were reached during the pre-cultivation periods. *B. amyloliquefaciens* reached an OD_600_ of 1.6 after 15 h. An OD_600_ of 2.3 was obtained for *C. coralloides* after 15 h. *P. fallax* reached OD_600_ 1.3 after 72 h. *R. jostii* reached an OD_600_ of 7.4 after 43 h. *S. griseochromogenes* was cultured for 15 h. The biomass measurement by OD_600_ of this bacterium was not possible because of clotted growth. In the main culture, various culture conditions were investigated. In all cases but the addition of supernatant (see Section 2.3.), 10% inoculum was used in 90 mL fresh medium in baffled 500 mL flasks and cultivated on an orbital shaker at 150 rpm and 30 °C. The cultivation period was 3 days for *B. amyloliquefaciens* and *S. griseochromogenes* and 7 days for *C. coralloides*, *P. fallax,* and *R. jostii*. The growth curves of control group cultures are shown in the Supplementary Materials (Figure S1).

### 2.3. OSMAC Experiments 

The variations of culture conditions are summarized in Table 2. In total, every bacterium was tested in 30 conditions. Media recipes can be found in Tables S8–S13.

To generate the two biotic additives, culture supernatant and cell pellets, inducer microbes were cultivated according to the cultivation protocol for the inoculum described above. Here, 10 mL of inoculum and 10 mL of autoclaved and centrifuged or centrifuged and sterile-filtered supernatant of the inducer microbe were added to 80 mL of fresh medium. In the case of the pellet additive, 10 mL of inoculum and 200–500 µL of autoclaved cell pellet were re-suspended in fresh medium and added to 90 mL of fresh medium.

### 2.4. Preparation of Biotic Additives 

Bacterial cultures, which were used as biotic additives, were grown for 5 days at 30 °C in 20 mL medium. The cultures were transferred into sterile Falcon tubes under sterile conditions and centrifuged in a Sorvall™ RC 5B Plus centrifuge at 3795× *g* for 20 min at 4 °C. The supernatant was sterile-filtered and directly used as biotic additive as described in the previous section. The remaining cell pellet was resuspended in 1 mL of the medium used in the main culture for better pipetting properties and then used as biotic additive, as described.

### 2.5. Cell Removal 

After termination of the cultivation, cells were removed from the broth by centrifugation in a Sorvall™ Rc 5B Plus centrifuge (Thermo Fisher, Waltham, MA, USA) by centrifuging for 20 min at 3795× *g* and 4 °C. Subsequently, the supernatant was filtered through folded cellulose filters with a pore size between 8 and 12 µm (Sartorius Folded Filters, Grade: 3 hw, Dia: 185 mm, 65 g∙m^3^, Sartorius, Göttingen, Germany). The cell pellet was collected in the respective folded filters, dried at 70 °C for 24 h, and weighed after drying.

### 2.6. Extraction of Cell-Free Fermentation Broth 

The cell-free broth was extracted three times with the equivalent volume of ethyl acetate, which has previously been used as solvent in natural product extraction [42]. The solvent was then evaporated to dryness to gain a dry extract, which was further prepared for HPLC-MS measurement.

### 2.7. HPLC-MS Sample Preparation 

Here, 2 mL of methanol (LC-MS grade) was added to the dry extracts and the extracts were dissolved in the solvent via ultrasonic bath. The concentrated extract was then filtered through 0.45 µm nylon filters (Chromafil^®^ Xtra PA-45/13, Macherey&Nagel, Düren, Germany) directly into glass vials.

### 2.8. HPLC-MS/MS Measurement 

The utilized high-performance liquid chromatography-mass spectrometry (HPLC-MS) system comprised an Agilent Technologies 1260 Infinity HPLC system (Agilent Technologies, Santa Clara, CA, USA) and a Bruker Compact ESI-QTOF-MS System (Bruker, Billerica, MA, USA). The injected sample volume was 2 µL. The solvents used were ACN (solvent A) and H_2_O with 0.1% formic acid (solvent B) with a flow of 0.4 mL/min. The following HPLC method was employed: 0–10 min 5%–98% solvent A, 10–15 min 98% solvent A isocratic, 15–17 min 98%–5% solvent A, 17–20 min 5% solvent A at a temperature of 40 °C with a C18-column (100 × 2.6 mm NucleoShell RP18, Macherey&Nagel, Düren, Germany). Diode-array detection (DAD) was used with a setting from 205 nm to 400 nm. The mass spectrometer comprised an electrospray ion (ESI) source and a time-of-flight (TOF) analyzer and was used in positive mode. Negative mode measurements were omitted because preliminary experiments revealed that a significantly higher number of peaks was detected in positive mode. The ESI source was used with a nebulizing gas pressure of 4 bar, a drying gas flow of 12 L/min, a drying temperature of 220 °C, and a capillary voltage of 4500 V. A mass-to-charge-ratio (*m/z*) range from 100 to 850 was measured. Each sample was measured once. For some samples, Auto-MS2 experiments were conducted. For some specific mass features, multiple reaction monitoring (MRM) measurements were used, applying identical HPLC conditions.

### 2.9. Evaluation of HPLC-MS Data 

For the evaluation of HPLC-MS data, the Bruker software Compass Data Analysis 1.7 was used. The obtained data were evaluated to determine the number of new mass features as a result of the change in culture conditions. In addition, the specific search of mass features was conducted to identify SM compounds. A mass feature was composed of a molecular mass *M_r_* and the corresponding retention time t_R_. msConvert from the ProteoWizard software (ProteoWizard, Palo Alto, CA, USA) was used for the conversion of vendor format raw data to mzXML-format [43]. The tool MZmine 2.35 [44] was used to identify new mass features by comparison of obtained samples with control group samples and medium background as well as the background of additives where necessary, e.g., supernatants. The steps and settings for raw data conversion, mzXML-raw data preparation, and peak list processing can be found in the Supplementary Material (Tables S14–S16). Additionally, automated and manual refining of the obtained results was necessary to eliminate false positive hits. A strict intensity threshold of 10^5^ was set and mass features were only considered as new if they reached or surpassed that limit in both biological duplicates. The new mass features were compared to a list of known contaminants in mass spectrometry [45]. New mass features were numbered and named according to the producing strain (BaXY = *B. amyloliquefaciens*, CcXY = *C. coralloides*, PfXY = *P. fallax*, RjXY = *R. jostii*, SgXY = *S. griseochromogenes*). Predicted compounds were determined using the extracted ion chromatogram (EIC) function of the Compass Data Analysis software for [M + H]^+^, [M + NH_4_]^+^, [M + Na]^+^, and [M + K]^+^ adduct identification with an allowed deviation of 0.01 Da.

### 2.10. Utilized Databases 

For the comparison of fragmentation patterns generated through HPLC-MS2 experiments to database entries, GNPS library search was used in default mode, i.e., precursor ion mass tolerance = 2.0 Da, fragment ion mass tolerance = 0.5 Da, minimum number of matched peaks = 6, and score threshold = 0.7. The cosine score indicates similarity between MS2 spectra and ranges from 0 to 1 (0 = dissimilar spectra, 1 = identical spectra) Here, matches with a cosine of 0.7 or higher were considered [46]. For the comparison of the experimental data to in silico fragmentation patterns of candidate molecules, MetFrag was used in default mode (allowed deviation from neutral mass: 5 ppm or 0.001 mzabs, mode: [M + H]^+^, tree depth = 2) with PubChem as database.

## 3. Results and Discussion

### 3.1. Genome-Mining for Selection of Bacteria

**Figure 1:**
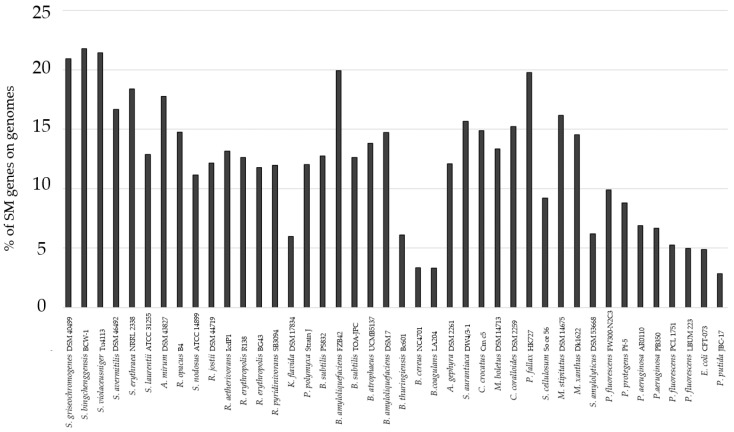
Percentage of genes relating to the entire number of mapped genes in the genome of the selected bacteria. The genome mining approach was carried out with antiSMASH [9]. A total of 42 bacteria were investigated for their potential to harbor silent biosynthetic gene clusters (BGCs) in their genome. The complete list of bacteria is presented in the supplement (Table S1). SM, secondary metabolite.

For an initial bioinformatics evaluation, 43 bacteria were considered with regard to biological safety, availability of deposited cultures, genome sequences, and literature coverage. The genomes of these bacteria were subjected to antiSMASH genome analysis. The set of bacteria included known SM producers and unexplored bacteria with respect to their biosynthetic potential. The bacteria and their NCBI accession numbers are summarized in Table S1. The genome analysis showed that the percentage of the bacterial genome encoding putative SM gene clusters varies greatly among the examined bacteria. On average, approximately 12% of the genome sequences of the selected bacteria are related to SM production. Some bacteria displayed an increased fraction of more than 20% of their genomes for SM production (Figure 1). 

Out of the 43 bacteria, we selected five strains that showed a high potential for the SM production. These bacteria either displayed a high percentage of SM biosynthesis pathway genes or possess interesting BGCs for polyketides, non-ribosomal peptides, and orphan BGCs. The selected bacteria were *S. griseochromogenes* DSM40499 with 20.92% BGCs on its genome, *R. jostii* DSM44719 with 12.17% BGCs on its genome, *C. coralloides* DSM2259 with 15.23% BGCs on its genome, *P. fallax* HKI727 with 19.08% BGCs on its genome, and *B. amyloliquefaciens* DSM7 with 14.72% BGCs on its genome. The total number of BGCs present on the genomes of the selected bacteria varies between 11 and 49 (Table 2, Tables S17 and S18, Figures S3–S6). Furthermore, only few of the predicted compounds were reported in the literature (Table 3).

### 3.2. Verification of AntiSMASH-Predicted SM Compounds 

The production of SM compounds, predicted by antiSMASH, was verified using a broad OSMAC approach. The experimental steps in the OSMAC approach included the cultivation of the selected bacteria under various conditions, the subsequent extraction of the culture supernatant, and HPLC-MS analyses to detect novel mass features corresponding to SM compounds. The chosen cultivation conditions varied in the composition of nutrient-rich media and chemically defined minimal media; PO_4_^3−^-, Fe^3+^-, and Mg^2+^-limitation; and the addition of organic solvents as well as different biotic additives. Overall, seven predicted products were detected in the generated extracts. The production of five compounds was solely detectable in the OSMAC samples and they were not detected in control group samples. Control group means the cultivation of the bacteria in their DSMZ-recommended, nutrient-rich media (NB medium for *B. amyloliquefaciens*, SP medium for *C. coralloides*, MD1 medium for *P. fallax*, TSB medium for *R. jostii*, and GYM medium for *S. griseochromogenes*). This contrasts with bacillaene (Figure S7) and desferrioxamine B (Figure S8), which were detected in control group samples and various generated OSMAC extracts (Table 4, Table S14).

In extracts obtained from *B. amyloliquefaciens*, the predicted products surfactins, bacillibactin, and bacillaene were detected. The production of surfactins by *B. amyloliquefaciens* was activated by various OSMAC conditions, including minimal media, Fe^3+^-limitation, and the addition of organic solvents (Table S19, Figure S9). Contrary to the broad OSMAC activation of the surfactins, bacillibactin (PubChem ID: 125349) was only detected in Fe^3+^-limited GMS extracts. This seems reasonable because of its nature of being a siderophore. Siderophores are secreted in Fe^3+^-starvation conditions and facilitate its uptake [53] (Figure S10). Bacillaene was detected in extracts from several culture conditions as well as in the control group grown in NB medium (Figure S7). The compound’s peak area in the EIC was most prominent in OSMAC conditions like Fe^3+^-limitation, GMS medium, and the addition of sterile-filtered supernatant of *P. fallax*. Its peak area decreased in extracts isolated from OSMAC experiments with LB medium and the addition of solvents.

In extracts obtained from OSMAC cultivations of *P. fallax*, the predicted compounds nostophycin and myxochelin A were detected. Nostophycin was identified using MS2-fragmentation and subsequent comparison with an in silico fragmentation pattern using MetFrag and the corresponding PubChem entry (PubChem ID: 101945102) (Figures S11 and S12, Table S20). Nostophycin was detectable in extracts from *P. fallax* grown under Fe^3+^- and Mg^2+^-limitation, in GMS medium, and in media with high concentrations of ethanol or toluene. The addition of toluene resulted in the highest peak intensity of nostophycin. The compound myxochelin A was detected under diverse OSMAC conditions (Figure S13). The production of myxochelin A by *P. fallax* was already verified in OSMAC conditions using MD1 medium [50], which could be confirmed here. Although a high number of BGCs can be found in the genome of *C. coralloides* and *R. jostii*, none of the predicted compounds were detected in the respective extracts.

In spite of our broad OSMAC screening, the number of detected compounds in comparison with the predicted compounds remained low. Our findings indicate that additional factors aside from the number of BGCs on the genome and OSMAC conditions should be taken into account for the evaluation of the biosynthetic potential of an organism. Firstly, the success of finding new SMs through OSMAC approaches will remain challenging and a broad range of different OSMAC conditions are necessary to enable the activation of various BGCs in the genome. The low number of detected and verified compounds indicate the OSMAC conditions, required for the activation of the predicted BGCs, have not been met. New conditions and multifactorial designs of OSMAC screenings provide a straightforward solution to find the required inducing conditions for the activation of silent BGCs. Interestingly, the detected SMs were produced under culture conditions, which were previously less reported in the literature. The overview of producing conditions in Table S14 shows that, especially, the culture conditions with limited prior literature coverage are responsible for the activation of the detected and predicted SMs. Secondly, a high similarity of BGCs in the selected bacteria with database entries also did not imply that the predicted compounds were produced by the bacteria under any of the tested OSMAC conditions. Indeed, high sequence similarity to database BGCs alone is not a good indicator to evaluate the biosynthetic potential. One explanation of our findings might be that the BGCs are dysfunctional, caused by certain detrimental variations and other mutations present in the BGC sequence. Hence, an in-depth analysis of BGCs including the structural integrity of detected BGCs and of the not yet annotated orphan BGCs is necessary aside from sequence similarity predicted by antiSMASH in order to better evaluate the biosynthetic potential of the selected bacteria.

### 3.3. Influence of Culture Conditions on the Number of New Mass Features

**Figure 2:**
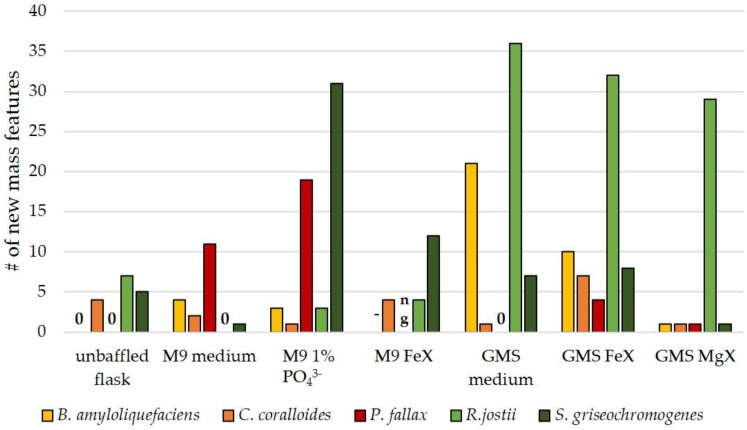
Number of new mass features in extracts of minimal media experiments, limitation experiments, and unbaffled flask experiments (all other experiments were conducted in baffled flasks). The ‘-’ indicates that the condition was not tested with this bacterium; a ‘0’ refers to no new mass features; and in the conditions marked with ‘ng’, bacteria did not grow. Every OSMAC condition was tested twice. The new mass feature had to be present in both biological duplicates. The mass feature signal had to surpass an intensity of 10^5^ [a.u.] to be considered for the number of new mass features. Therefore, this diagram only shows the number of reproducibly detected mass features in the duplicate cultivation.

Aside from the identified SM compounds, several novel mass features were detected under the chosen OSMAC conditions. The number of new mass features was mainly dependent on the OSMAC conditions. In fact, we found an increased number of mass features in infrequently applied OSMAC conditions, namely minimal media and limitation experiments. Figure 2 shows the number of new mass features in PO_4_^3−^-limitation (1% of amount of phosphate salts of original M9 medium recipe used), Fe^3+^-limitation, and Mg^2+^-limitation in M9 and GMS medium.

An increased number of mass features was detected in PO_4_^3−^-limitation experiments. This relates to the common assumption that high PO_4_^3−^-concentrations act as repressors of SMs, whereas a limited PO_4_^3−^-concentration is more preferable for secondary metabolism [14,35]. Masuma et al. showed that phosphate reduction enhanced antibiotic production in *Streptomyces rosa* [54]. *S. griseochromogenes* and *P. fallax* are more susceptible to the PO_4_^3−^-limitation than the other tested strains, as demonstrated by the high numbers of new mass features obtained under this condition. *R. jostii* reacted strongly to any variation of GMS medium with up to 36 new mass features.

The Fe^3+^-limitation clearly resulted in an increased number of new mass features. Interestingly, the number of new mass features varied considerably between M9 FeX and GMS FeX cultures. Four new mass features were detected in Fe^3+^-limited *R. jostii* cultures in M9 medium versus 32 new mass features in Fe^3+^-limitation on GMS medium, and their identities were completely different. The same pattern could be seen in *C. coralloides* and *S. griseochromogenes* cultures (Table 5). This observation shows that the formation of novel compounds is not solely a result of Fe^3+^-limitation, but is also dependent on other components present in the minimal medium. The greatest differences are the phosphate concentrations with significantly lower amounts in GMS medium; the presence and absence, respectively, of several trace elements; as well as further compounds such as amino acids, vitamins, and salts. Especially, the availability of phosphate and trace elements might have influenced the (de)activation of several BGCs.

**Figure 3:**
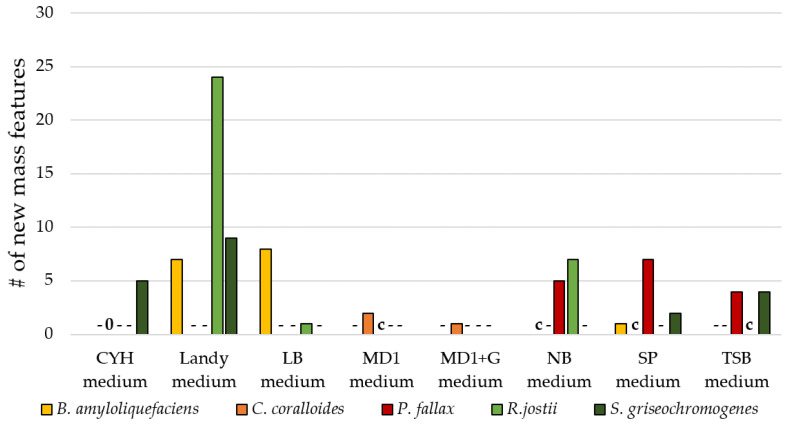
Number of new mass features in extracts of nutrient-rich media experiments. The ‘-’ indicates not tested with this bacterium, the ’0’ related to no new mass features compared with the control group ‘c’. Any new mass feature had to be present in both biological duplicates and its MS signal had to surpass an intensity of 10^5^ to be considered for the number of new mass features presented in this diagram. Therefore, this diagram only shows the number of reproducibly detected mass features.

The use of nutrient-rich media is the most frequent OSMAC approach for SM discovery and BGC activation [55]. In nutrient-rich media, different complex nutrient sources are used, which presumably influence secondary metabolism [56]. Some chosen media possess trace elements (MD1, MD1+G) or amino acids (Landy), some possess several complex nutrient sources (CY/H), and others possess two complex components (NB). In this study, we tested a total of eight different nutrient-rich media, which have previously been used in OSMAC-based media screenings [57,58]. Three different media per strain were tested (Figure 3).

Owing to the compositional differences of the nutrient-rich media, we expected several new mass features in the extracts of the bacteria grown on various media. Figure 3 shows that new mass features indeed appeared in the OSMAC media screening. Especially, the Landy extracts showed a high numbers of new mass features. An explanation for the increased number of mass features was the difference in composition of the Landy medium compared with the media used for the control group of each bacterium. While the control group media are based on complex carbon and nitrogen sources such as casitone, yeast extract, or meat extract, the main components of Landy medium are glucose, glutamic acid, and phenylalanine. This difference in composition explains the more pronounced change in the metabolite profile [55]. Still, this observation is surprising, because glucose is known to interfere with secondary metabolism in many strains via carbon catabolite repression [56]. Ruiz et al. give an overview of SMs underlying carbon catabolite repression like actinorhodin in *Streptomyces coelicolor*. However, SMs that are not under the control of carbon catabolite repression are known as well (bacilysin from *Bacillus subtilis)* [59]. Other carbon sources can also have interfering or non-interfering effects on SMs, such as glycerol interfering with cephalosporin production in *Cephalosporium acremonium*, but not with simocyclinones in *Streptomyces antibioticus* Tü6040. Lactose and starch have not been reported as interfering with the production of any SMs. As *B. amyloliquefaciens*, *R. jostii*, and *S. griseochromogenes* produced several new mass features when grown in Landy medium, the produced mass features seem to not underlie carbon catabolite repression.

Following the impact of nutrient-rich media on SM production, the effect of solvents was evaluated. The addition of solvents for the elicitation of SMs from bacteria has not yet been sufficiently reported. Among the few publications dealing with the influence of solvents, ethanol was most frequently used. Apart from the study by Chen et al. from the year 2000 [20], Doull et al. and Jakeman et al. examined the influence of ethanol shock on jadomycin B production in *Streptomyces venezuelae* [22,60], and Chatterjee et al. investigated the effect of very low concentrations of ethanol on the viability and growth recovery of *Staphylococcus aureus* [61]. Therefore, cultivations with higher ethanol concentrations (0.5/1/3/6 Vol%) were used in this study. Additionally, we included dimethyl sulfoxide (DMSO), toluene (Tol), and acetonitrile (ACN) in the OSMAC screening. DMSO was already used by Chen et al. and affected antibiotic production in *Bacillus* and *Streptomyces* strains [20]. To the best of our knowledge, neither acteonitrile nor toluene have previously been investigated regarding SM elicitation. It has previously been reported that immediate addition of ethanol has a positive effect on SM production, while too late addition results in decreased SM production [22,60]. In our experiments, the organic solvents were hence immediately added to the main cultures.

**Figure 4:**
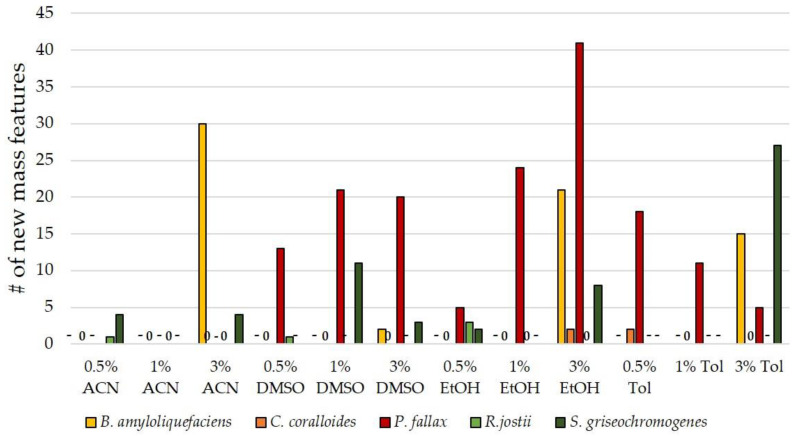
Number of new mass features in extracts of solvent supplementation experiments. Entries marked with ‘-’ were not tested and ‘0’ indicates no new mass features with this bacterium. Any new mass feature had to be present in both biological duplicates and its MS signal had to surpass an intensity of 10^5^ to be considered for the number of new mass features presented in this diagram. Therefore, this diagram only shows the number of reproducibly detected mass features. DMSO, dimethyl sulfoxide; ACN, acetonitrile; Tol, toluene; EtOH, ethanol.

The analysis of the extracts of OSMAC conditions in the presence of solvents showed heterogeneous results across the selected strains. While *C. coralloides* was unsusceptible to almost all tested solvents and concentrations, *P. fallax* displayed up to 41 new mass features in the presence of DMSO, ethanol, and toluene (Figure 4). *S. griseochromogenes* responded with new mass features to all tested organic solvent additives. *B. amyloliquefaciens* reached between 15 and 30 new mass features in response to toluene, ethanol, and acetonitrile, but only produced 2 new mass features when supplemented with DMSO. This confirms the observation of Chen et al. that the solvent effects are highly dependent on the microbe’s stress tolerance [20]. Different tolerance mechanisms have been proposed for ethanol and DMSO, among them the action on the translational level and change of membrane structure, but no definite statement can be given at this point [21,61]. In sum, the OSMAC approach with solvent addition is a promising route to discover new SMs. Particularly, more organic solvents should be tested for their potential to increase the number of mass features as a proxy of new SMs.

**Figure 5:**
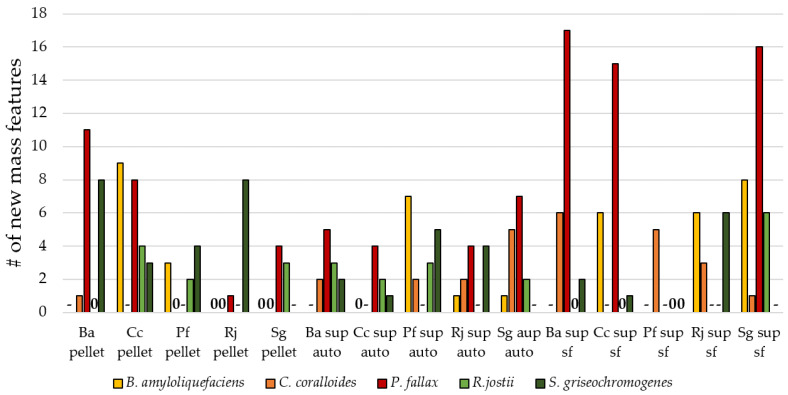
Number of new mass features in extracts of biotic additive experiments. A ‘-’ indicates not tested with this bacterium, while ‘0’ relates to no new mass features. The bacteria are indicated as follows: Ba = B. *amyloliquefaciens*, Cc = *C. coralloides*, Pf = *P. fallax*, Rj = *R. jostii*, and Sg = *S. griseochromogenes.* Any new mass feature had to be present in both biological duplicates and its MS signal had to surpass an intensity of 10^5^ to be considered for the number of new mass features presented in this diagram. Therefore, this diagram only shows the number of reproducibly detected mass features.

It has been shown in manifold research papers that co-cultivation is a productive way of exploiting the biosynthetic potential of microbes [62]. Several small molecules produced by one organism are bioactive in other organisms. For example, acyl homoserine lactones are actively involved in proteobacteria signaling and peptides are known as signaling molecules in gram-positive bacteria [63]. Moreover, the addition of supernatants and culture extracts, or the use of heat-killed cells, were successful in generating SMs [27,33]. However, the use of such biotic additives has not been widely exploited to mimic the presence of signal molecules and surface proteins to trigger a metabolic response in microorganisms. In order to study the influence of biotic additives on SM production, sterile-filtered and autoclaved supernatants obtained from various bacterial strains were added to bacterial cultures. New mass features were generated in both experimental setups in all tested strains (Figure 5). In general, we observed that pretreatment of bioactive additives had a minor influence on the number of mass features. The addition of sterile-filtered supernatant did not generally lead to a higher number of new mass features compared with the autoclaved supernatant. It is likely that most signaling molecules were sufficiently temperature-stable during the autoclaving process.

In the case of *S. griseochromogenes* cultures, two new mass features were detected when supplemented with sterile-filtered supernatant from *B. amyloliquefaciens* and another two mass features when supplemented with its autoclaved supernatant. These new mass features were not the same for sterile-filtered and autoclaved supernatant. The compounds Sg255 (Peak #255) originated from the *S. griseochromogenes* culture, Sg345 from cultivation with sterile-filtered supernatant, and Sg358 and Sg359 from cultivation with autoclaved supernatant. Sg255 was also produced when cultures were supplemented with 0.5% toluene or sterile-filtered supernatant of *R. jostii*. Sg345 was also produced when an autoclaved cell pellet of *R. jostii* was added. On the contrary, Sg358 and Sg359 were condition-specific and were only produced when autoclaved supernatant from *B. amyloliquefaciens* was added to *S. griseochromogenes* cultures.

In general, the supernatant types were equally well suited to generate new mass features. An exception was the OSMAC approach with *P. fallax*, with a clear benefit of the sterile-filtered supernatant. Here, we obtained an increased number of new mass features compared with all tested bacteria with added supernatants. The addition of autoclaved cell pellets also worked well for the generation of new mass features in *P. fallax*, *R. jostii*, and *S. griseochromogenes* cultures. Certain bacteria responded only to a specific bacterial pellet. For example, *C. coralloides* only reacted to the addition of *B. amyloliquefaciens* pellet and *B. amyloliquefaciens* only reacted to the myxobacteria *C. coralloides* and *P. fallax*. This observation might be due to the relationship between the known predatory myxobacteria and their prey, which often constitutes *Bacillus* species [64]. In comparison with the addition of organic solvents, the addition of the three types of biotic additives seems to generate new mass features in the selected bacteria more reliably.

To sum up, after data curation, 590 new mass features, which are likely to originate from secondary metabolism, from the five strains were obtained through OSMAC experiments. This number greatly surpasses the number of encoded BGCs and verifies the assumed biosynthetic potential of the selected bacterial strains. Table 6 summarizes the detected new mass features and the number of predicted BGCs in comparison with the genome size.

Apart from *C. coralloides*, the selected bacteria generated significantly more new mass features under the tested culture conditions than predicted. The results showed that a high number of predicted BGCs does not necessarily correspond to high biosynthetic potential in terms of a high number of new mass features, as evident when comparing, e.g., *B. amyloliquefaciens* and *C. coralloides* (Table 6). Overall, the selected bacteria produced many new mass features under the tested culture conditions and the selection of biosynthetically promising bacteria was thus successful. Additionally, it could be shown that the culture conditions with limited prior literature coverage—for example, addition of solvents, biotic additives, and limitations—produced many new mass features, which need to be further investigated.

### 3.4. Suitability of Culture Conditions for the Generation of New Mass Features 

**Figure 6:**
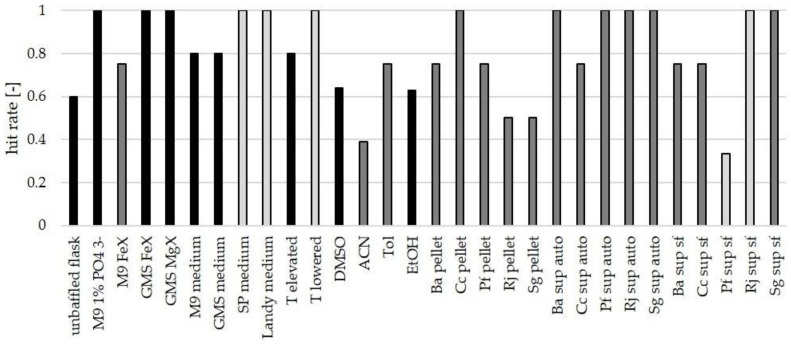
Hit rates for all tested culture conditions. Black bars: sample size *n* = 5 strains, dark grey bars: *n* = 4, light grey bars: *n* = 3.

Interestingly, the tested culture conditions were not equally successful in eliciting new mass features and the selected bacteria seem to be susceptible to different conditions. Overall, it was observed that all tested culture conditions are pleiotropic as they generated more than one new mass feature in at least one bacterial strain [65]. The authors hence agree with Bode’s statement that no “common rules for all microorganisms” can be developed from OSMAC screenings and that “everything is allowed to find new natural products” as long as no complete understanding of secondary metabolism and its regulation is achieved [15]. However, our findings demonstrate that some culture conditions are more promising than others and constitute good starting points for future screenings. Therefore, we evaluated the selected culture conditions regarding their general capability to provoke new mass features from the considered bacteria. This capability is termed hit rate and is defined as the number of strains generating new mass features in response to one culture condition over the number of tested strains. The results show that 13 of the 30 tested culture conditions led to the detection of new mass features in all strains. In other words, 43% of the tested OSMAC conditions led to a hit rate of 1 (Figure 6).

The addition of several biotic additives (cell pellet of *C. coralloides*, autoclaved supernatants of all strains, but *C. coralloides*) and some limitation experiments (PO_4_^3−^-limitation on M9 medium and Fe^3+^- and Mg^2+^-limitation on GMS medium) have reliably provoked new mass features from all tested microorganisms. These OSMAC conditions are recommended as starting points for future screenings with other bacteria. 

**Figure 7:**
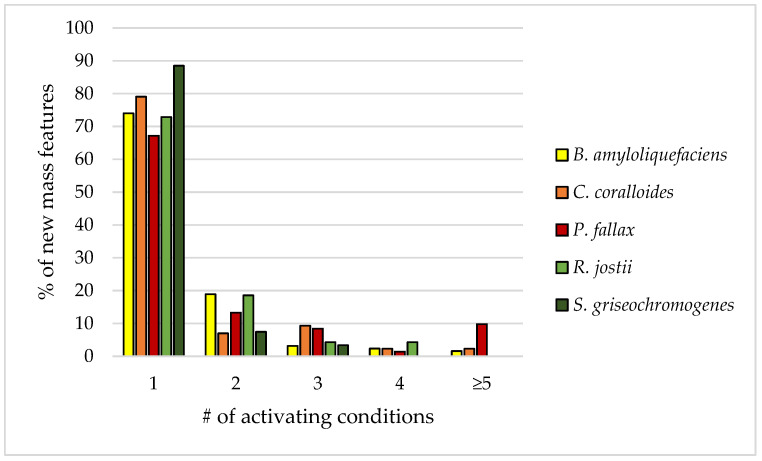
Percentage of new mass features that are activated by a certain number of culture conditions.

Our analysis also shows that, in most tested strains, more than 70% of the generated new mass features were produced under exactly one of the tested culture conditions (condition-specific). Between 7 and 19% of the new mass features were generated under two culture conditions, and even less under three or more culture conditions (Figure 7).

In general, we postulate that a broad screening of many culture conditions with a high hit rate applied to carefully selected and promising bacterial strains is a recommendable way for the generation of new mass features.

### 3.5. Putative Annotation of New Mass Features

The analysis of HPLC-MS data resulted in detailed lists of new mass features for each investigated sample containing *M_r_*, t_R_s, and culture conditions (Tables S16–S20). To assess the likelihood of the new mass features being SMs, they were categorized into groups based on their physicochemical properties. The categorization was carried out by comparing the *M_r_* and t_R_ of the mass features to published data of 56 primary metabolites and 128 bacterial SMs (Figures S15 and S16). The 590 new mass features were evaluated regarding their intensity and peak area with heat maps for prioritization (Tables S21–S25, Figures S17–S19). The top 5% regarding peak area were subjected to HPLC-MS2 measurements to obtain their compound-specific fragmentation patterns. Identified mass features, their corresponding compounds, and means of identification are listed in Table 7. The corresponding producing conditions are listed in Table S26. Matches to reference compounds comprised matching t_R_s and [M + H]^+^. The mass deviation was less than 5 ppm in all cases, but myxochelin A (Δppm = 10), and is specified in the Supplementary Material.

The production of bacillibactin (Ba58), myxochelin A (Pf336), and desferrioxamine B (Sg117 derivative, Sg130 Al-complex) was already described above, as their BGCs were predicted by antiSMASH. Following the predicted compounds, various novel compounds could be assigned.

Ba3 has been identified as the cyclic dipeptide cyclo-(Tyr-Pro) (Figure S20) and has not been reported in the literature as a product from *B. amyloliquefaciens* DSM7. It is produced under various culture conditions: Mg^2+^-limitation, and two different nutrient-rich media. Strains from *B. subtilis*, are generally regarded as producers of cyclic peptides, which have antimicrobial activity against gram-negative strains [66,67].

The mass features named Ba8, Ba9, and Ba10 have been putatively matched with iturins A-4, A-6, and A-8 (Figures S21–S24). Interestingly, no BGC related to iturin was detected by antiSMASH, although it is a common antifungal SM of *B. amyloliquefaciens* strains [68]. A detailed analysis of the *B. amyloliquefaciens* DSM7 genome independent of antiSMASH conducted in 2011 revealed an iturin BGC and a follow-up study is the only literature report of iturin production by this exact strain [69,70]. In the study by Borriss et al., iturin A was detected in DSM7 cultures grown in Landy medium [70], while we detected the compound in LB cultures. Because our antiSMASH genome analysis revealed an orphan NRPS BGC on the genome of *B. amyloliquefaciens* DSM7, this may be the BGC responsible for iturin A synthesis.

Cc42 has been identified as nocardamin, a cyclic desferrioxamine B derivative, and thus a siderophore [71] (Figure S25), This compound has not yet been reported in the literature as a product from *C. coralloides*. Nocardamin has only been detected in actinomycetes, mostly cultures of *Streptomyces*, and has, to the best of our knowledge, never before been reported for any proteobacterium. Our antiSMASH analysis has not revealed the nocardamin BGC. Nevertheless, the *C. coralloides* genome is not fully annotated and harbors many orphan BGCs, which could potentially code for the nocardamin BGC.

Streptomycetes are known producers of desferrioxamine siderophores, which include nocardamin (=desferrioxamine E) [72]. Different desferrioxamine derivatives have been detected in our *S. griseochromogenes* OSMAC cultures as well as in the control group. It has been observed that a change in culture conditions influences the distribution of produced derivatives (data not shown). Mass feature Sg117, desmethyl-enyl nocardamin, was only detected in the iron-deprived M9 culture (M9 FeX) and the complex of desferrioxamine B with aluminum was only detected in cultures supplemented with DMSO (Figures S26 and S27).

## 4. Conclusions

The biosynthetically promising and fully sequenced bacterial strains *B. amyloliquefaciens* DSM7, *C. coralloides* DSM2259, *P. fallax* HKI727, *R. jostii* DSM77419, and *S. griseochromogenes* DSM40499 were selected for OSMAC experiments based on genome mining, availability, and literature research to activate their predicted BGCs. The five selected bacteria were cultured under 30 different OSMAC conditions and 308 extracts were obtained, resulting in 590 new mass features. Among all tested bacteria, five BGCs could be activated using OSMAC conditions: surfactin variants and bacillibactin from *B. amyloliquefaciens* extracts, myxochelin A and putatively nostophycin from *P. fallax* extracts, and putatively albaflavenone from *S. griseochromogenes* extracts. Additionally, bacillaene and desferrioxamine B were verified in cultures of *B. amyloliquefaciens* and *S. griseochromogenes*, respectively. Because these two compounds were already present in the control group samples, their BGCs were not activated. Still, some predicted BGCs remained silent under the tested culture conditions, some even despite 100% sequence similarity. These results demonstrate that high sequence similarity alone is not an adequate indicator for the biosynthetic potential of strains. Furthermore, the genomes of the investigated bacteria possess high numbers of orphan BGCs. Thus, the biosynthetic potential of the investigated bacteria has not yet been fully elucidated and holds more to discover.

The culture conditions with limited literature coverage have successfully contributed to the activation of predicted BGCs as well as to the generation of new mass features. Some culture conditions provoked the production of new mass features more reliably than others. Furthermore, more than 70% of the generated new mass features were condition-specific. The biological potential of SM production by varying culture conditions is obviously far from being exhausted, as demonstrated by this broadly chosen OSMAC approach. Most of the tested bacteria surpass the expected number of novel mass features. The high number of provoked new mass features, e.g., 147 new peaks in *S. griseochromogenes* DSM40499 extracts, validates the high biosynthetic potential of the selected strains. From these observations, we conclude the following statements: Firstly, PO_4_^3-^-limited M9 medium, Fe^3+^-limited GMS medium, and several biotic additives are promising starting points for future screenings. Secondly, a broad screening with many tested culture conditions and a small number of carefully selected strains is a promising approach for the search for novel bacterial SMs because of the condition-specificity of mass features. If a broader screening leads to more mass features provoked by several conditions, statistical analysis tools like principal component analysis (PCA) could be employed to study the silent cluster activation mechanisms in more detail [73]. The large number of new mass features presents us with new challenges, namely the elucidation of the novel compounds and the investigation of their biological properties. The potential to find new promising molecules with interesting properties among these substances is enormous.

## Figures and Tables

**Table 1:** Summary of new pharmaceutically active compounds obtained by altering culture conditions in one strain many compounds (OSMAC) screening approaches.

Strain	Natural Product	Altered Conditions	Properties	Reference
*Sphaeropsidales* sp. F-24’707	42 cladospirones	variation of media	antibacterial and antifungal	[16]
*Streptomyces* sp. MBT27	quinazolinone A and B	variation of carbon sources	anti-inflammatory, antitumor, antimicrobial, and anti-fungal properties	[17]
*Aspergillus* sp. LS34	9 new compounds	solid rice medium and potato dextrose medium	cytotoxic activity against cancer cell lines/active against pathogenic *Staphylococcus aureus*	[18]

**Table 2:** Summary of tested OSMAC culture conditions used in this study. Tryptic Soy Broth (TSB), Lysogeny Broth (LB), Glucose-Yeast-Malt (GYM), Nutrient Broth (NB), Glucose Minimal Salt (GMS).

Category	Tested Conditions
culture vessel	unbaffled flask instead of baffled flask
nutrient-rich media	3 per bacterium out of SP medium, TSB medium, CY/H medium, MD1 medium, MD1+G medium, LB medium, GYM medium, NB medium, Landy medium, Luria medium
minimal media	M9 medium, GMS medium
limitations	Mg^2+^-, PO_4_^3−^- and Fe^2+^ limitation (named MgX [no Mg^2+^-salts], FeX [no Fe^3+^-salts], 1% PO_4_^3−^ [1% of amount of phosphate salts in original recipe])
organic solvents	acetonitrile (ACN), dimethyl sulfoxide (DMSO), ethanol (EtOH), toluene (Tol) at 0.5/1/3/6 Vol %
biotic additives	autoclaved supernatant, sterile-filtered supernatant, autoclaved cell pellet of *B. amyloliqufaciens*, *C. coralloides*, *P. fallax*, *R. jostii*, and *S. griseochromogenes*

**Table 3:** Summary of the biosynthetic potential of the five selected strains based on the genome mining approach with antiSMASH. The percentage of detected biosynthetic gene clusters (BGCs) in the genome, the total number of BGCs, and the number of known secondary metabolite (SM) compounds of these selected bacteria are shown in the table.

Strain	Genome Size [Mbp]	% BGCs	Number of BGCs	Known Compounds
*B. amyloliquefaciens*	3.98	14.7	11	surfactin [47], bacillaene [48], fengycin [47], and bacillibactin [49] from *B. amyloliquefaciens* FZB42
*C. coralloides*	10.08	15.2	34	-
*P. fallax*	10.77	19.1	33	myxochelin [50] from *P. fallax* HKI727
*R. jostii*	7.89	12.2	18	rhodochelin [51] from *R. jostii* RHA1
*S. griseochromogenes*	10.76	20.9	49	blasticidin [52] from *S. griseochromogenes* (not further specified) heterologously expressed in *S. lividans*

**Table 4:** Summary of detected compounds from the number of searchable compounds based on the predicted non-orphan BGCs in the five selected bacteria. These compounds were identified in the analyzed extracts of the bacteria. Mass-based identification was carried out with the extracted ion chromatogram (EIC) function of the Compass Data Analysis software with 0.01 Da tolerance and under consideration of the isotopic profile. Deviations of the measured *m*/*z* from the calculated [M + H]^+^ in ppm are indicated in the table. A comparison of experimental MS2-spectra with in silico fragmentation patterns generated via MetFrag was used for bacillibactin and nostophycin. Additionally, the fragmentation pattern of bacillibactin was compared to literature data [49]. Compounds in bold were only found in extracts of OSMAC experiments, but not in the control group. The producing conditions for each compound can be taken from Table S19. Control group means the cultivation of the bacteria in their DSMZ-recommended, nutrient-rich media (NB medium for *B. amyloliquefaciens*, SP medium for *C. coralloides*, MD1 medium for *P. fallax*, TSB medium for *R. jostii*, and GYM medium for *S. griseochromogenes*).

Strain	Number of BGCs According to AntiSMASH	Number of Searchable Compounds	Detected Compounds (% Sequence Similarity)	Means of Identification		
				Mass-Based	MS2- Fragmentation (MetFrag)	Reference Compound
***B. amyloliquefaciens***	**11**	**6**	surfactin (82%)			✓ Δppm = 2.2 (C15) Δppm = 4.9 (C14)
			bacillibactin (100%)		✓ (and [49]) Δppm = 1.4	
			bacillaene (100%)	✓ Δppm = 0.7		
*C. coralloides*	34	12	-			
*P. fallax*	33	22	**myxochelin A (75%)**			✓ Δppm = 10.1
			**nostophycin (18%)**		✓ Δppm = 7.0	
*R. jostii*	18	13	-			
*S. griseochromogenes*	49	38	desferrioxamine B (100%)			✓ Δppm = 4.6
			**albaflavenone (100%)**	✓ Δppm = 38.8		

**Table 5:** Comparison of new mass features produced by *C. coralloides* and *S. griseochromogenes* on M9 FeX medium and GMS FeX medium. The mass features’ properties (*m/z* and t_R_) can be found in the Supplementary Material.

Strain	New Mass Features on M9 FeX	New Mass Features on GMS FeX
*C. coralloides*	Cc243, Cc244, Cc249, Cc250	Cc22, Cc181, Cc186, Cc187, Cc189, Cc193, Cc210
*S. griseochromogenes*	Sg115, Sg117, Sg119, Sg120, Sg135, Sg198, Sg199, Sg201, Sg203, Sg206, Sg207, Sg214	Sg96, Sg97, Sg101, Sg103, Sg104, Sg106, Sg107, Sg109

**Table 6:** Genome size, numbers of predicted BGCs, and numbers of detected new mass features per strain.

Strain	*B. amyloliquefaciens*	*C. coralloides*	*P. fallax*	*R. jostii*	*S. griseo-chromogenes*
Genome size [Mbp]	3.98	10.08	10.76	7.89	10.77
Number of predicted BGCs	11	34	33	18	49
Number of new mass features in extracts	127	35	143	138	147

**Table 7:** Results of fragmentation pattern analysis of selected new mass features. The new mass features were identified by the fragmentation pattern library of the web-based mass spectrometry ecosystem Global Natural Products Social Molecular Networking (GNPS), MetFrag or a reference compound. Evidence is given in the Supplementary Material. A summary of the experimental fragmentation pattern is given in Table S27. The level of confidence for the identification of all compounds is given in Table S28. The ID is composed of the abbreviation of the producer strain (Ba = *B. amyloliquefaciens*, Cc = *C. coralloides*, Pf = *P. fallax*, Sg = *S. griseochromogenes*) and the number of the detected new mass features.

ID	*m/z*	t_R_ [min]	Proposed Compound	Means of Identification		
				GNPS	MetFrag	Reference Compound
Ba3	261.12	4.5	cyclo (Tyr-Pro)	✓		✓ Δppm = 0.7
Ba8	1057.57	7.2	iturin A-4	✓	✓	
Ba9	1071.58	7.6	iturin A-6	mass-based		
Ba10	1085.6	7.9	iturin A-8		✓	
Ba58	883.26	6.1	bacillibactin	comparison to fragmentation pattern from literature, Δppm = 1.4		
Cc42	601.36	4.7	nocardamin	✓		✓ Δppm = 4.4
Pf336	405.16	5.4	myxochelin A			✓ Δppm = 10.1
Sg117	587.35	4.5	desmethyl enyl nocardamin Δppm = 0 (GNPS)	✓		
Sg130	585.36	4.9	desferrioxamine B + Al Δppm = 13 (GNPS)	✓		

## Data Availability

Data is contained within the article or Supplementary Material.

## Supplementary Materials

The following are available online at https://www.mdpi.com/2218-273X/11/2/193/s1, Figure S1: Growth curves of selected bacterial under control group conditions, Figure S2: antiSMASH predictions for *Bacillus amyloliquefaciens* DSM7, Figure S3: antiSMASH predictions for *Rhodococcus jostii* DSM44719, Figure S4: antiSMASH predictions for *Streptomyces griseochromogenes* DSM40499, Figure S5: antiSMASH predictions for *Corallococcus coralloides* DSM2259, Figure S6: antiSMASH predictions for *Pyxidicoccus fallax* HKI727, Figure S7: Extracted Ion Chromatograms (EICs) of singly and double charged of putative bacillaene from *B. amyloliquefaciens* cultures grown on LB medium and corresponding MS-spectrum, Figure S8: Comparison of chromatograms (EICs) and MS-spectra of desferrioxamine B reference compound and EIC of *S. griseochromogenes* control group sample, Figure S9: (a) Comparison of chromatograms of surfactin reference compound and Extracted Ion Chromatograms (EICs) from sample *B. amyloliquefaciens* grown at 40 °C. (b) Comparison of MS-spectra of surfactin reference compound and sample *B. amyloliquefaciens* grown at 40 °C, Figure S10: Experimental fragmentation pattern of bacillibactin with 45eV, Figure S11: Total Ion Chromatogram (TIC) and fragmentation pattern of putative nostophycin in sample *P. fallax* 6 Vol% toluene, Figure S12: MetFrag results of *in silico* fragmentation of PubChem entry #101945102 compared with experimental MS2 spectrum of putative nostophycin, Figure S13: Comparison of chromatograms and MS-spectra of myxochelin A reference compound and EIC of sample *P. fallax* M9 1% PO43-, Figure S14: Extracted Ion Chromatogram and corresponding MS-spectrum of putative albaflavenone from oxygen limitation sample of *S. griseochromogenes* culture, Figure S15: Comparison of physicochemical data of bacterial primary and secondary metabolites taken from PubChem, Figure S16: Visualization of employed HPLC method and correlation of logP values and ACN percentage at t_R_, Figure S17: Heat map for new mass features of *B. amyloliquefaciens* DSM7 and utilized color code, Figure S18: (a) Heat map of *S. griseochromogenes* DSM40499; (b) Heat map of *C. coralloides* DSM2259. Rows = mass features, columns = producing conditions, Figure S19: (a) Heat map of *P. fallax* HKI727; (b) Heat map of *R. jostii* DSM44719. Rows = mass features, columns = producing conditions, Figure S20: Comparison of Extracted Ion Chromatograms (EICs) and MS spectra of cyclo-(tyr-pro) reference compound and cyclo-(tyr-pro) in *B. amyloliquefaciens* sample grown at 40 °C, Figure S21: Extracted Ion Chromatograms of putative iturins A4, A-6 and A-8 from *B. amyloliquefaciens* sample grown on LB medium and Total Ion Chromatogram of MS2-experiment from the same sample, Figure S22: MS2-spectra of putative iturin A-4, A-6 and A-8 masses in *B. amyloliquefaciens* sample grown on LB medium, Figure S23: MetFrag settings used for the verification of MS2-fragments of putative iturin A-8 with corresponding PubChem entry #10866068, Figure S24: MetFrag explanations of fragments obtained through MS2-analysis of mass 1085.59 (putative iturin A-8) in *B. amyloliquefaciens* DSM7 sample grown on LB medium, Figure S25: Comparison of Extracted Ion Chromatograms (EICs) and MS spectra of nocardamin reference compound and nocardamin in *C. coralloides* sample grown in an unbaffled flask, Figure S26: Results of GNPS search of desmethyl enyl nocardamin MS2-spectrum (mirror match and overview), Figure S27: Results of GNPS search of desferrioxamine B + Al MS2-spectrum (mirror match and overview). Table S1: NCBI Accession numbers of candidate strains examined with antiSMASH, Table S2: List of chemicals, chemical formulae, and supplier, Table S3: Composition of NB medium, Table S4: Composition of SP medium, Table S5: Composition of MD1 medium, Table S6: Composition of TSB medium, Table S7: Composition of GYM medium, Table S8: Composition of CY/H medium, Table S9: Composition of LB medium, Table S10: Composition of MD1+G medium, Table S11: Composition of Landy medium, Table S12: Composition of Glucose Minerals Salts (GMS) medium, Table S13: Composition of M9 medium and 1% PO43- M9 medium, Table S14: Settings for vendor format raw data conversion with msConvert according to GNPS website instructions, Table S15: Steps for raw data preparation with MZmine 2.35, Table S16: Steps for peak list processing with MZmine 2.35 with t_R_ = retention time, Table S17: Numbers of BGCs of different natural product classes present on the genomes of selected strains, Table S18: Color code used for natural product classes in genome maps, Table S19: Overview of compounds and corresponding producing conditions from selected strains. Table S20: m/z of fragments generated through MS2 experiment of sample *P. fallax* 6 Vol% toluene and corresponding intensities used for *in silico* fragmentation of PubChem entry #101945102 for verification of nostophycin, Table S21: List of new mass features detected in extracts from *B. amyloliquefaciens* DSM7, Table S22: List of new mass features detected in extracts from *C. coralloides* DSM2259, Table S23: List of new mass features detected in extracts from *P. fallax* HKI727, Table S24: List of new mass features detected in extracts from *R. jostii* DSM44719, Table S25: List of new mass features detected in extracts from *S. griseochromogenes* DSM40499, Table S26: Overview of new mass features, proposed compounds, and corresponding producing conditions from selected strains, Table S27: Summary of fragmentation patterns and collision energies (CE) of compounds identified among the new mass features, Table S28: Summary of detected compounds and level of confidence for their identification.
