# Supplementary material for: Triaging of Culture Conditions for Enhanced Secondary Metabolite Diversity from Different Bacteria
Source: Biomolecules. 2021 Jan 30;11(2):193. doi: 10.3390/biom11020193 (PMC7911347; doi:10.3390/biom11020193)
Supplement: Supplementary file 1 [file biomolecules-11-00193-s001.pdf]

Supplementary Information

Table S1. NCBI Accession numbers of candidate strains examined with antiSMASH.

Strain	NCBI Accession #	Strain	NCBI Accession #
Bacillus cereus NC4701	AP007209	*Myxococcus xanthus* DK 1622	CP000113
Bacillus thuringiensis Bc60	CP015150	*Corallococcus coralloides DSM2259*	NC_017030
Bacillus subtilis TOA-JPC	NZ_CP011882	*Myxococcus stipitatus* DSM14675	CP004025
Bacillus subtilis PS832	NZ_CP010053	*Pyxidicoccus fallax HKI727*	Obtained from Prof. Nett's group of Technical Biology, TU Dortmund University
Bacillus atrophaeus UCMB5137	CP011802	*Streptomyces nodosus* ATCC14899	CP009313
Bacillus amyloliquefaciens DSM7	NC_014551	*Rhodococcus erythropolis* BG43	CP011295
Pseudomonas putida JBC17	CP029693	*Rhodococcus pyridinivorans* SB3094	NC_023150
Escherichia coli CFT-073	AE014075	*Rhodococcus jostii DSM44719*	NZ_FNTL01000004
Pseudomonas fluorescens LBUM223	NZ_CP011117	*Streptomyces laurentii* ATCC31255	AP017424
Pseudomonas protegens Pf-5	NC_04129	*Rhodococcus aetherivorans* IcdP1	NZ_CP011341
Streptomyces rapamycinicus NRRL5491	CP006567	*Streptomyces avermitilis* DSM46492	NZ_KQ948581
Sorangium cellulosum So ce56	AM746676	*Streptomyces griseochromogenes DSM40499*	NZ_CP016279
Paenibacillus polymyxa strain J	NZ_CP015423	*Actinosynnema mirum* DSM43827	NC_013093
Saccharopolyspora erythraea NRRL2338	NC_009142	*Streptomyces bingchenggensis* BCW-1	NC_016582
Pseudomonas aeruginosa AR0110	CP029745	*Pseudomonas aeruginosa* PB350	CP025055
Streptomyces violaceusniger Tu 4113	CP002994	*Rhodococcus opacus* B4	NC_012522

Rhodococcus erythropolis R138	NZ_CP007255	*Kribbella flavida* DSM 17836	NC_013729
Bacillus amyloliquefaciens FZB42	NC_009725	*Pseudomonas fluorescens* str. PCL1751	NZ_CP010896
Pseudomonas fluorescens str. FW300-N2C3	NZ_CP012831	*Bacillus coagulans*	CP025437
Melittangium boletus DSM 14713	CP022163	*S. aurantiaca* DW4/3-1	CP002271
Archangium gephyra DSM 2261	CP011509	*S. amylolyticus DSM 53668*	NZ_CP011125
Chondromyces crocatus Cm c5	CP012159		

Table S2. List of chemicals, chemical formulae, and supplier. Chemicals were used in the purest form available.

**Chemical**	**Chemical formula**	**Supplier**	**Chemical**	**Chemical formula**	**Supplier**
BactoPeptone	-	Beckto Dickinson	magnesium sulfate heptahydrate	$MgSO_4$ x 7 H_2O	Carl Roth
meat extract	-	Fluka	dipotassium-phosphate	K_2HPO_4	Carl Roth
raffinose D(+) pentahydrate	$C_{18}H_{32}O_{16}$ x 5 H_2O	Sigma Aldrich	calcium chloride dihydrate	$CaCl_2$ x 2 H_2O	Carl Roth
sucrose D(+)	$C_{12}H_{22}O_{11}$	Carl Roth	vitamin B12	$C_{63}H_{88}CoN_{14}O_{14}P$	Carl Roth
galactose D(+)	$C_6H_{12}O_6$	Carl Roth	EDTA	$C_{10}H_{16}N_2O_8$	Carl Roth
soluble starch	$(C_6H_{10}O_5)n$	Carl Roth	iron (II) sulfate heptahydrate	$FeSO_4$ x 7 H_2O	Carl Roth
BactoCasitone	-	Beckto Dickinson	zinc sulfate heptahydrate	$ZnSO_4$ x 7 H_2O	AppliChem
boric Acid	H_3BO_3	Carl Roth	manganese (II) cloride tetrahydrate	$MnCl_2$ x 4 H_2O	Carl Roth
cobalt (II) chloride hexahydrate	$CoCl_2$ x 6 H_2O	Carl Roth	copper (II) chloride dihydrate	$CuCl_2$ x 2 H_2O	Carl Roth
nickel (II) chloride dihydrate	$NiCl_2$ x 6 H_2O	Alfa Aesar	sodium molybdate dihydrate	$MoNa_2O_4$ x 2 H_2O	Carl Roth
peptone from soybean	-	Carl Roth	D (+) glucose	$C_6H_{12}O_6$	Carl Roth
sodium	NaCl	Carl Roth	Bacto yeast	-	Beckto

chloride			extract		Dickinson
malt extract	-	Carl Roth	HEPES	$C_8H_{18}N_2O_4S$	Carl Roth
tryptone	-	Carl Roth	L-glutamic acid	$C_5H_9NO_4$	AppliChem
potassium chloride	KCl	Carl Roth	sodium nitrate	$NaNO_3$	Carl Roth
potassium dihydrogen phosphate	KH_2PO_4	Carl Roth	thiamine-HCl	$C_{12}H_{17}ClN_4OS$ x HCl	Carl Roth
L-phenyl-alanine	$C_9H_{11}NO_2$	Carl Roth	L-proline	$C_5H_9NO_2$	Carl Roth
manganese (II) sulfate hydrate	$MnSO_4$ x H_2O	Carl Roth	disodium hydrogen phosphate	Na_2HPO_4 x 2 H_2O	AppliChem
copper (II) sulfate pentahydrate	$CuSO_4$ x 5 H_2O	Carl Roth	ammonium chloride	NH_4Cl	Carl Roth
ethyl acetate	$C_4H_8O_2$	VWR	methanol	CH_4O	Carl Roth
acetonitrile	C_2H_3N	Carl Roth	dimethyl sulfoxide	C_2H_6OS	Fisher Scientific
ethanol	C_2H_6O	Merck	toluene	C_7H_8	Fisher Scientific
surfactin	$C_{53}H_{93}N_7O_{13}$ $C_{52}H_{89}N_7O_{13}$	Santa Cruz Biotechno-logy	myxochelin A	$C_{20}H_{24}N_2O_7$	Biomol
cyclo-(tyr-pro)	$C_{14}H_{16}N_2O_3$	Carbo-synth Limited	nocardamin	$C_{27}H_{48}N_6O_9$	Biomol
desferrioxa-mine B	$C_{25}H_{48}N_6O_8$	Sigma			

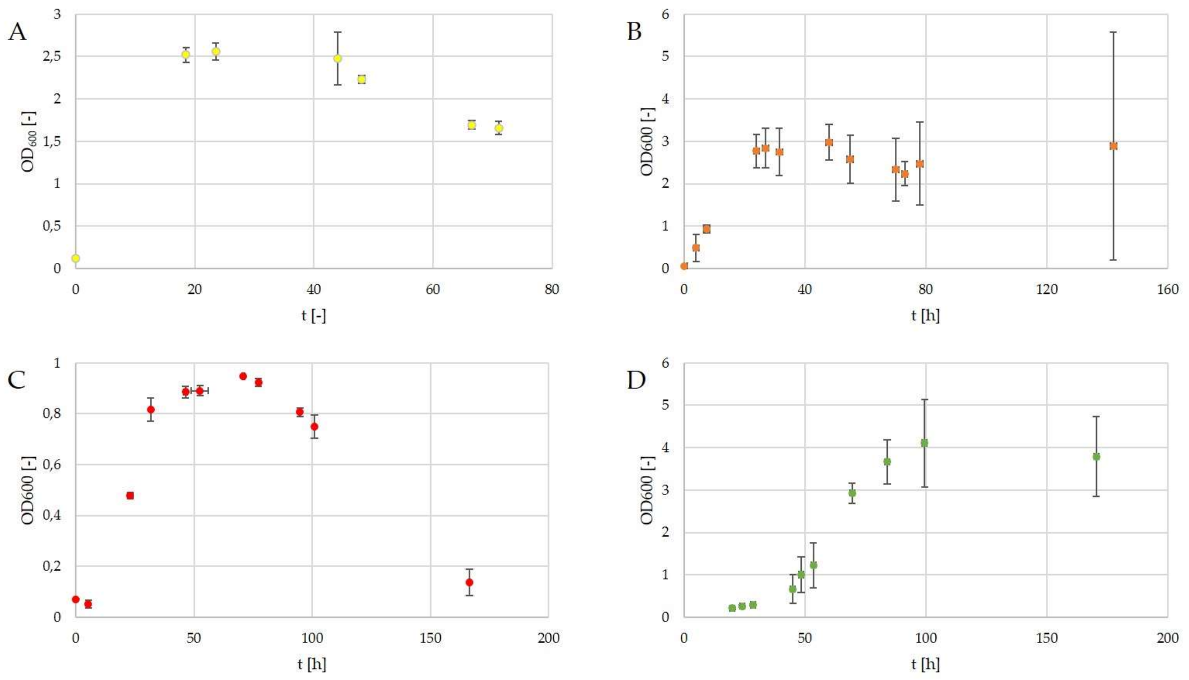

Figure S1. Growth curves of selected bacterial under control group conditions. A-*B. amyloliquefaciens* on NB medium at 30°C, B- *C. corallloides* on SP medium at 30°C, C- *P. fallax* on MD1 medium at 30°C, D- *R. jostii* on TSB medium at 28°C.

Table S3. Composition of NB medium. Final pH = 7.0 [1].

**components**	**amount [g·L^{-1}]**
peptone from meat	5.0
meat extract	3.0

Table S4. Composition of SP medium. Final pH = 7.4 [2].

**components**	**amount [g·L^{-1}]**
raffinose	1.0
sucrose	1.0
galactose	1.0
soluble starch	5.0
Bacto® Casitone	2.5
MgSO4 x 7 H_2O	0.5
K_2HPO_4	0.25

Table S5. Composition of MD1 medium. Final pH = 7.0. Add 0.5 mg·L^{-1} vitamin B12 [3].

**components**	**amount [g·L^{-1}]**
Bacto® Casitone	3.0
$CaCl_2$ x 2 H_2O	0.7
MgSO4 x 7 H_2O	2.0
Trace Element Solution SL4	1 mL
**Trace Element Solution SL4**	**amount [g·L^{-1}]**
EDTA	0.8
$FeSO_4$ x 7 H_2O	0.2
$ZnSO_4$ x 7 H_2O	0.01
$MnCl_2$ x 4 H_2O	0.003
H_3BO_3	0.03
$CoCl_2$ x 6 H_2O	0.02
$CuCl_2$ x 2 H_2O	0.001
$NiCl_2$ x 6 H_2O	0.002
Na_2MoO_4 x 2 H_2O	0.003

Table S6. Composition of TSB medium. Final pH = 7.3 [4].

**components**	**amount [g·L^{-1}]**
Bacto® Casitone	17.0
peptone from soy	2.5
D-(+)-glucose	5.0
NaCl	3.0
K_2HPO_4	3.0

Table S7. Composition of GYM medium. Final pH = 7.2 [5].

**components**	**amount [g·L^{-1}]**

yeast extract	4.0
malt extract	10.0
D-(+)-glucose	4.0

Table S8. Composition of CY/H medium. Solutions A and B are to be autoclaved separately. 1 mL of each solution B and C will be added to solution A. Final pH = 7.4 [6].

**components**	**amount [g·L^{-1}]**
**solution A**	
Bacto® Casitone	1.5
yeast extract	1.5
starch	4.0
soy flour	1.0
D-(+)-glucose	1.0
$CaCl_2$ x 2 H_2O	1.0
MgSO4 x 7 H_2O	0.5
HEPES	11.9
**solution B**	
EDTA-iron	0.8 g
distilled water	100.0 ml
**solution C**	
vitamin B_{12}	0.05 g
distilled water	100.0 ml

Table S9. Composition of LB medium. Final pH = 7.0 [7].

**Components**	**amount [g·L^{-1}]**
tryptone	10.0
yeast extract	5.0
NaCl	10.0

Table S10. Composition of MD1+G medium. Final pH = 7.4.

**Components**	**amount [g·L^{-1}]**
MD1 recipe	
D-(+)-Glucose	2.2

Table S11. Composition of Landy medium. Final pH = 7.0 [8].

**Components**	**amount [g·L^{-1}]**
D-(+)-Glucose	20.0
L-glutamic acid	5.0
L-phenylalanine	2.0
$MgSO_4$ x 7 H_2O	0.5
KCl	0.5
KH_2PO_4	1.0
1% $FeSO_4$	15 µL/L
1% $MnSO_4$	500 µL/L
1% $CuSO_4$	16 µL/L

Table S12. Composition of Glucose Minerals Salts (GMS) medium. Final pH = 7.0. (No longer available at DSMZ.)

**Components**	**amount [g·L^{-1}]**
$NaNO_3$	2.0
K_2HPO_4	0.5
$MgSO_4$ x 7 H_2O	0.2
$MnSO_4$ x 5 H_2O	0.02
$FeSO_4$ x 7 H_2O	0.02
$CaCl_2$ x 7 H_2O	0.02
D-(+)-Glucose	5.0

Table S13. Composition of M9 medium and 1% PO_4^{3-} M9 medium. Final pH = 7.4 [9].

**components**	**amount [mL]**
1 M $MgSO_4$	1.0
0.1 M $CaCl_2$	1.0
1 M thiamine-HCl x 2 H_2O	1.0
D-(+)-glucose	2.01 g

L-proline	20.0 mg
distilled water	900.0
salt solution	100.0
**salt solution for M9 medium**	
Na_2HPO_4	6.0 g
KH_2PO_4	3.0 g
NH_4Cl	1.0 g
NaCl	0.5 g
distilled water	100.0
**salt solution for M9 medium 1% PO_4^{3-}**	
Na_2HPO_4	0.06 g
KH_2PO_4	0.03 g
NH_4Cl	0.01 g
NaCl	0.005 g
distilled water	100.0

Table S14. Settings for vendor format raw data conversion with msConvert according to GNPS website instructions [10].

Parameter	Setting
output format	mzXML
binary encoding precision	32 bit
write idex	✓ (check)
TPP compatibility	✓ (check)
use zlib compresion	uncheck
Filters	Peak Picking with algorithm = vendor and MS Level 1-

Table S15. Steps for raw data preparation with MZmine 2.35.

Input	Step	Settings	Output
raw data, mzXML-format	Peak Detection -> Mass Detection	MS level: 1<br>Mass Detector: Centroid<br>Noise level: 1.0E4	mass list
mass list	Peak Detection -> Chromatogram	MS level: 1<br>Min time span: 0.01	peak lists, suffix: chromatograms

	Builder	min<br>Min height: 3.0E4<br>*m/z* tolerance: 0.0 *m/z* or 20 ppm	

Table S16. Steps for peak list processing with MZmine 2.35 with t_R = retention time.

Input	Step	Settings	Output
peak lists, suffix: chromatograms	Peak Detection -> Chromatogram Deconvolution	Algorithm: Baseline cut-off	peak lists, suffix: chromatograms deconvoluted
peak lists, suffix: chromatograms deconvoluted	Isotopes -> Isotopic peaks grouper	m/z tolerance: 0.0 m/z or 20 ppm<br>t_R tolerance: 0.1 min<br>Maximum charge: 3<br>Representative isotope: lowest *m/z*	peak lists, suffix: chromatograms deconvoluted deisotoped
peak lists, suffix: chromatograms deconvoluted deisotoped	Alignment -> Join Aligner	*m/z* tolerance: 0.0 *m/z* or 20 ppm<br>Weight for *m/z*: 75<br>t_R tolerance: 0.1 min<br>Weight for t_R : 25	aligned peak list
aligned peak list	Filtering -> Peak list rows filter	Minimum peaks in a row: 2<br>remove rows that match all criteria	aligned peak list, suffix: filtered
aligned peak list, suffix: filtered	Filtering -> Peak list rows filter	t_R : 1.0-11.0 min<br>keep rows that match all criteria	aligned peak list, suffix: filtered filtered

Table S17. Numbers of BGCs of different natural product classes present on the genomes of selected strains. Some BGCs were not clearly identified and were labeled as two classes by antiSMASH. Those BGCs have been counted twice. Therefore, the total number of BGCs does not necessarily equal the sum of listed gene clusters. PKS = polyketides, NRPs = non-ribosomal peptides, PKs-NRPs = PKs-NRPs-hybrid.

Natural product class	# BGCs on DSM7 genome	# of BGCs on DSM2259 genome	# of BGCs on HKI727 genome	# of BGCs on DSM44719 genome	# BGCs on DSM40499 genome
PKs	2	3	7	2	7
NRPs	3	4	8	11	3

PKs-NRPs	2	8	9	0	8
Terpenes	2	4	1	2	8
Lantipeptides	4	5	0	0	6
Bacteriocins	0	5	5	2	7
Other	1	5	3	3	14
Total	**11**	**34**	**33**	**18**	**49**
Orphan clusters	**5**	**22**	**11**	**5**	**11**

Table S18. Color code used for natural product classes in genome maps.

Color	Natural Product Class
	Non-ribosomal peptide (NRPS)
	Siderophore
	Lantipeptide
	Bacteriocin
	Polyketides (PKS)
	Terpenes
	PKS-NRPS-hybrids
	Ectoine
	Other group
	lassopeptide

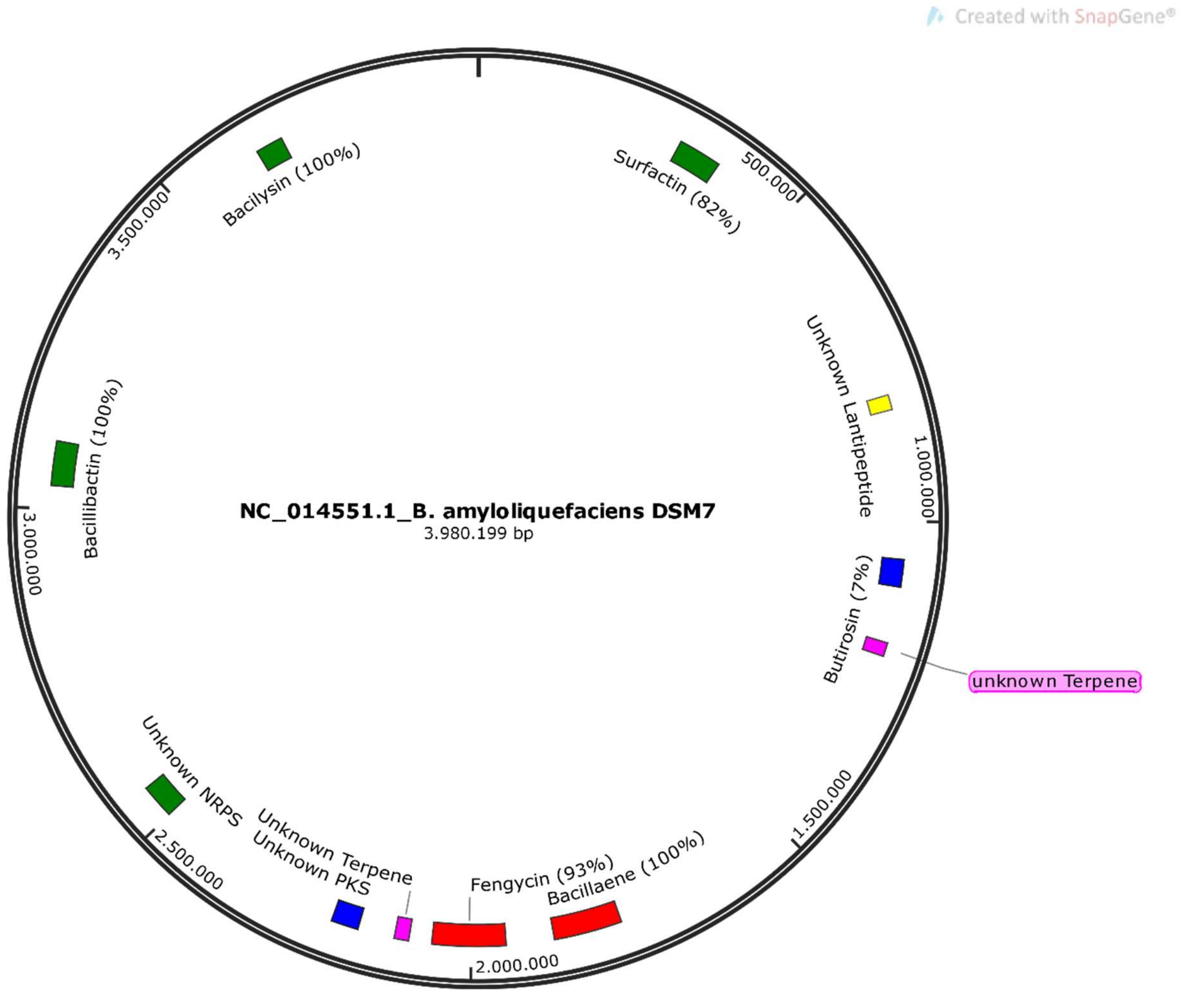

Figure S2. antiSMASH predictions for *Bacillus amyloliquefaciens* DSM7.

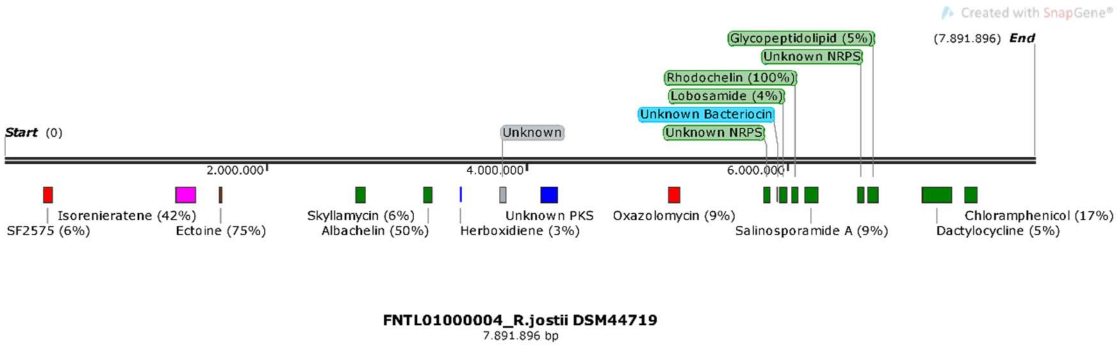

Figure S3. antiSMASH predictions for *Rhodococcus jostii* DSM44719.

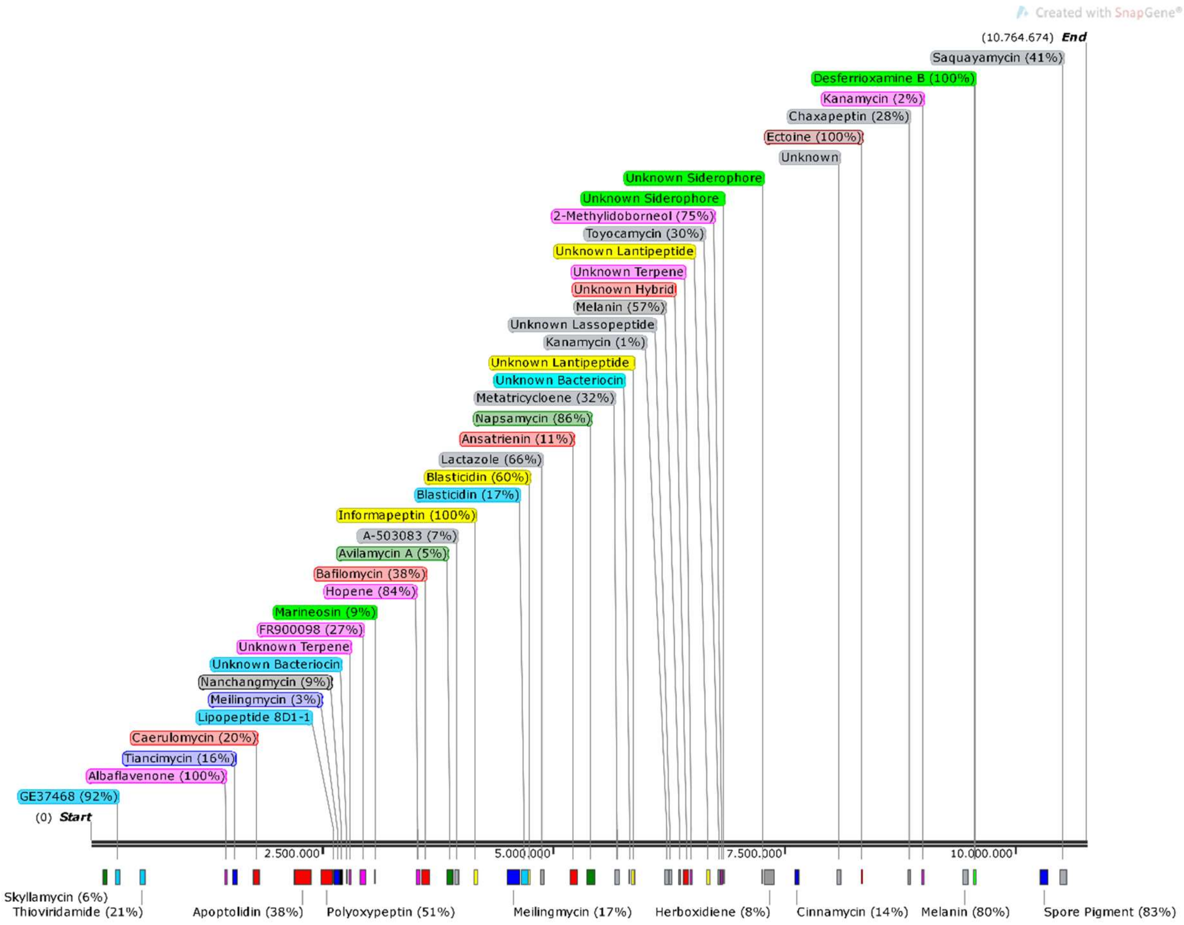

Figure S4. antiSMASH predictions for *Streptomyces griseochromogenes* DSM40499.

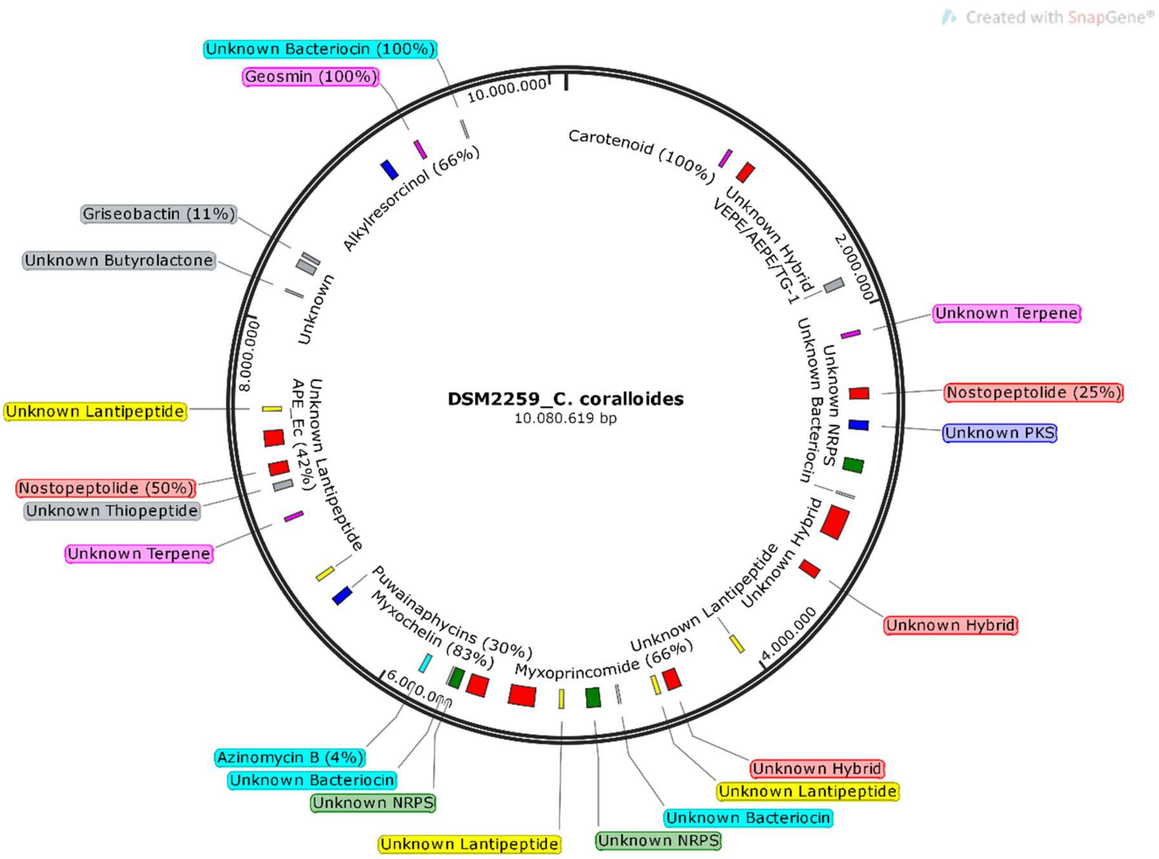

Figure S5. antiSMASH predictions for *Corallococcus coralloides* DSM2259.

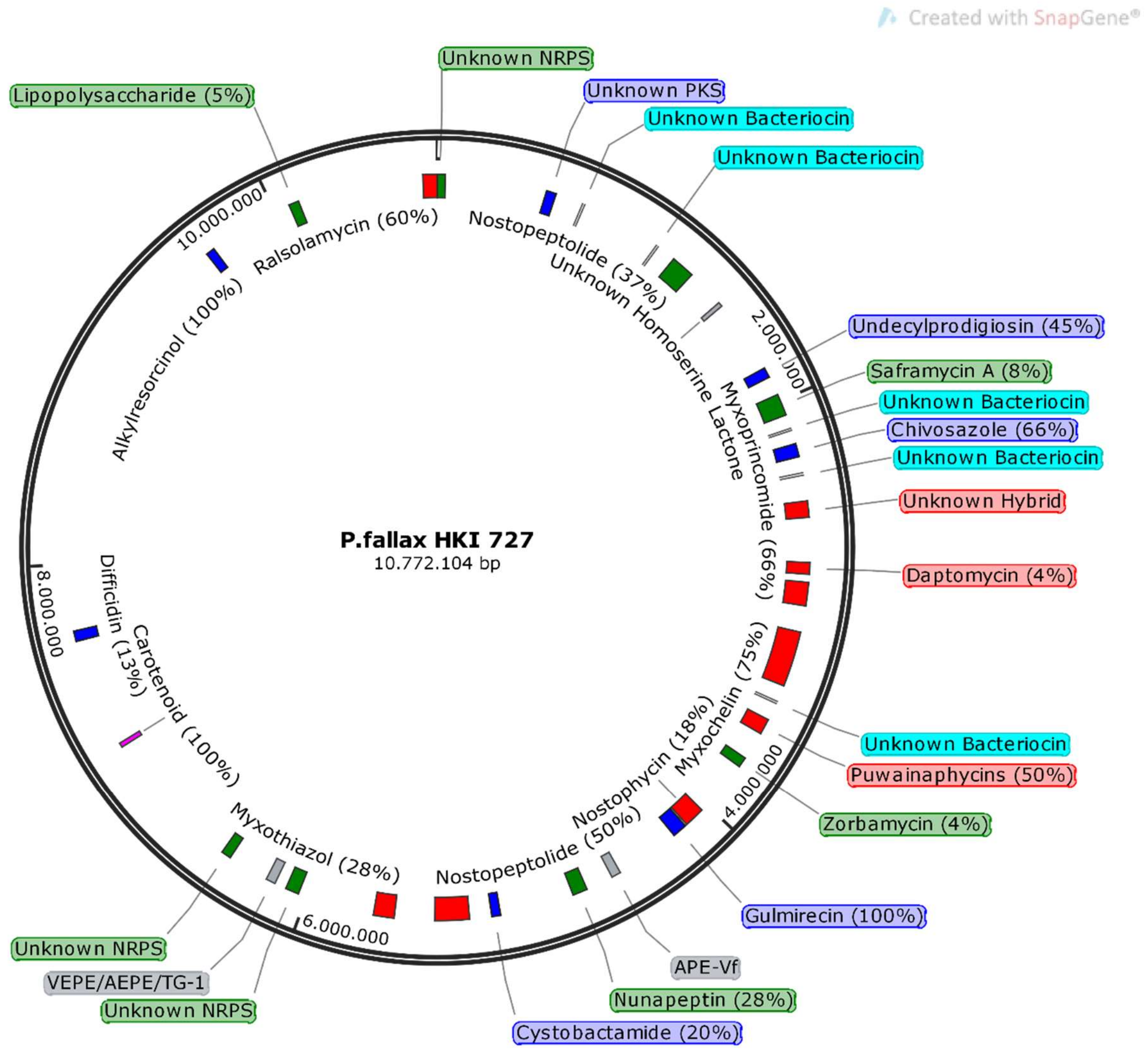

Figure S6. antiSMASH predictions for Pyxidicoccus fallax HKI727.

Table S19. Overview of compounds and corresponding producing conditions from selected strains. Bold print indicates that the compounds were not detected in control group samples.

Strain	Detected compound (% similarity)	Producing conditions
B. amyloliquefaciens	**surfactin** (82%)	40°C, GMS medium, Fe^{3+}-limited GMS medium (GMS FeX), 3% EtOH, 3% Tol, addition of sterile-filtered supernatant of *P. fallax*
	**bacillibactin** (100%)	Fe^{3+}-limited GMS medium (GMS FeX),
	putative bacillaene (100%)	control, LB medium, 40°C, Fe^{3+}-limited GMS medium (GMS FeX), 3% EtOH, 3% Tol, addition of sterile-filtered supernatant of *P. fallax*
P. fallax	**myxochelin A** (75%)	NB medium, SP medium, M9 medium, M9 medium with 1% PO_4^{3-}, 25°C, 35°C,

		0.5%/1%/3% DMSO, 0.5%/1% EtOH, addition of *C. coralloides* or *S. griseochromogenes* pellet.
	**nostophycin** (18%)	GMS medium, Fe^{3+}-limited GMS medium (GMS FeX), Mg^{2+}-limited GMS medium (GMS MgX), 6% EtOH, 6% Tol
S. griseochromogenes	desferrioxamine B (100%)	all tested conditions **BUT**: TSB medium, GMS medium, Mg^{2+}-limited GMS medium (GMS MgX), 6% ACN, addition of Tol, 8% EtOH
	putative **albaflavenone** (100%)	oxygen limitation, SP medium, GMS medium, Fe^{3+}-limited GMS medium (GMS FeX), Mg^{2+}-limited GMS medium (GMS MgX)

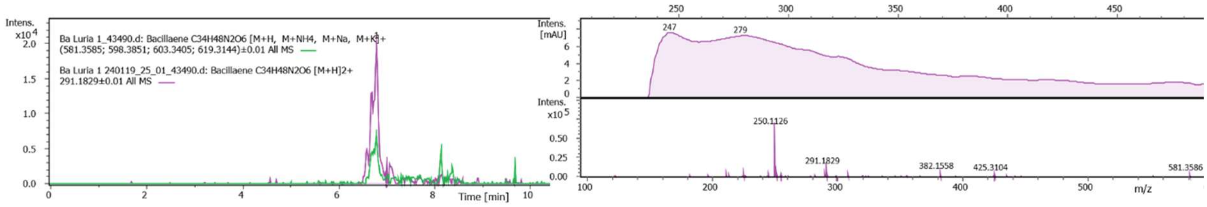

Figure S7: Extracted Ion Chromatograms (EICs) of singly and double charged of putative bacillaene from *B. amyloliquefaciens* cultures grown on LB medium and corresponding MS-spectrum. The UV spectrum is shown above the MS spectrum. Δppm = 0.7.

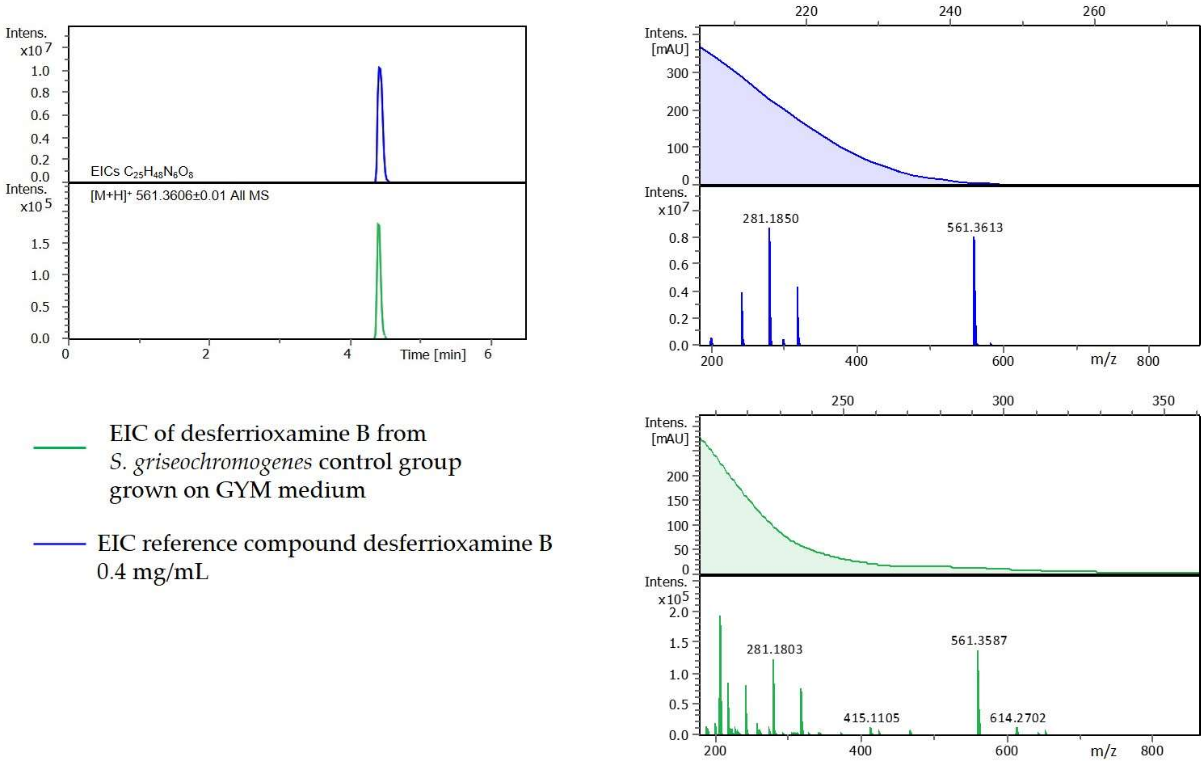

Figure S8. Comparison of chromatograms (EICs) and MS-spectra of desferrioxamine B reference compound (blue) and EIC of *S. griseochromogenes* control group sample (green). The UV spectrum is shown above the MS spectrum. Δppm = 4.6.

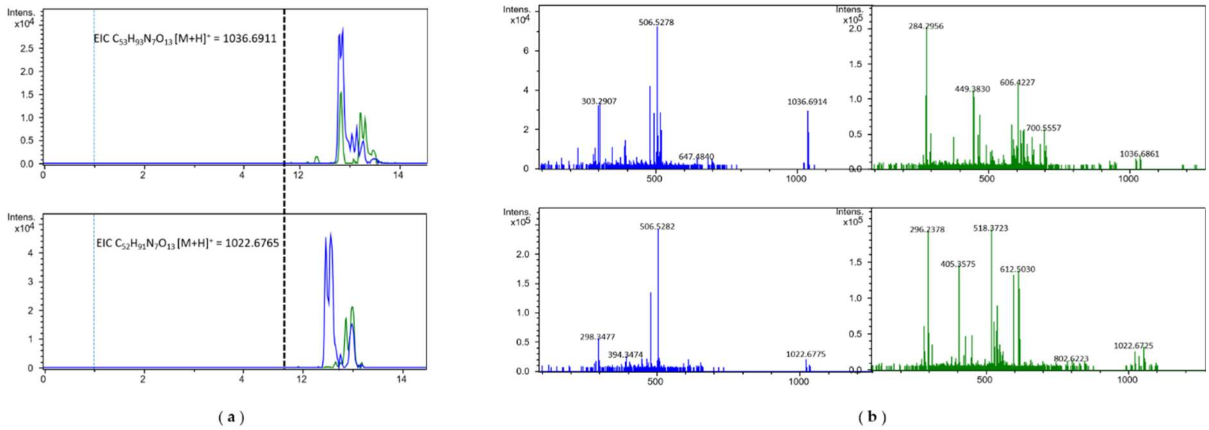

Figure S9. (**a**) Comparison of chromatograms of surfactin reference compund (contains C14- and C15-variant) (blue) and Extracted Ion Chromatograms (EICs) from sample *B. amyloliquefaciens* grown at 40°C (green). (**b**) Comparison of MS-spectra of surfactin reference compound and sample *B. amyloliquefaciens* grown at 40°C. Δppm = 4.9 for C14, Δppm = 2.2 for C15.

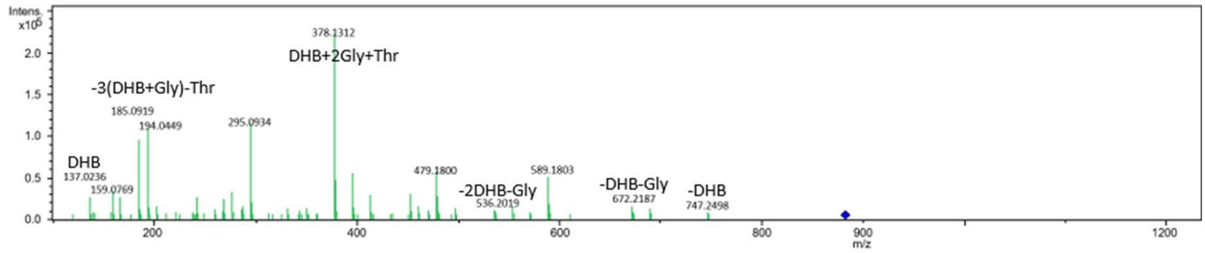

Figure S10. Experimental fragmentation pattern of bacillibactin with 45eV in accordance to [32]. Blue diamond marks the precursor molecule with *m/z* = 883.2424. Δppm = 1.4.

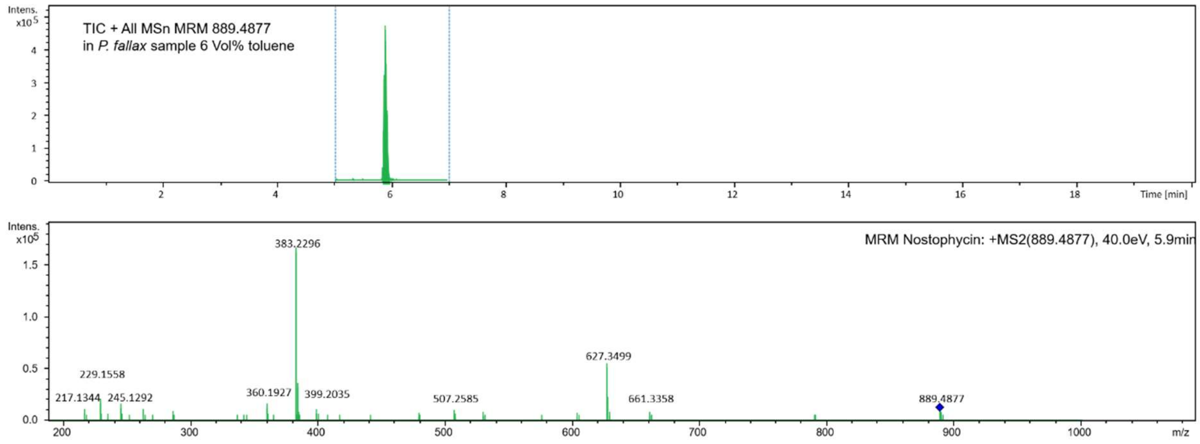

Figure S11. Total Ion Chromatogram (TIC) and fragmentation pattern of putative nostophycin in sample *P. fallax* 6 Vol% toluene. CE = 40eV. Δppm = 7.0.

Table S20. *m/z* of fragments generated through MS2 experiment of sample *P. fallax* 6 Vol% toluene and corresponding intensities used for *in silico* fragmentation of PubChem entry #101945102 for verification of nostophycin.

$m/z_{fragment}$	Intensity [%]
383.2296	100

627.3499	31.15
229.1558	10.26
360.1927	7.2
245.1292	7.2
263.1394	4.1
399.2035	4.0
217.1344	3.9
507.2585	3.5
286.1776	2.5
661.3358	2.0

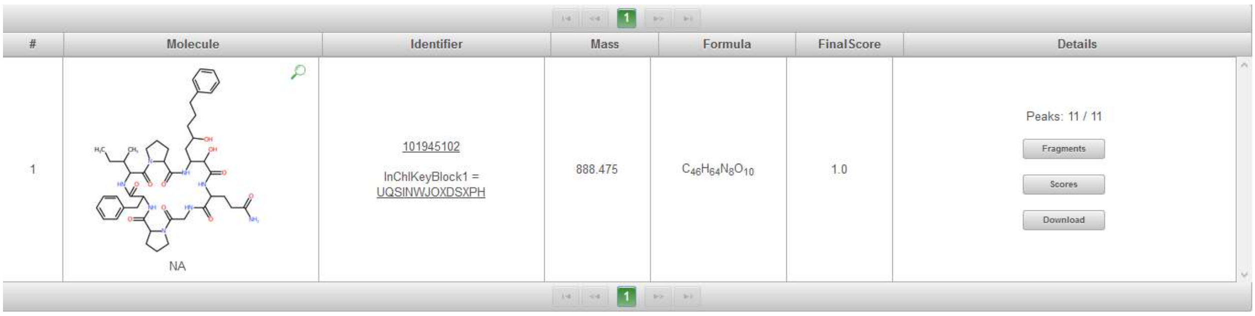

Figure S12. MetFrag results of in silico fragmentation of PubChem entry #101945102 compared with experimental MS2 spectrum of putative nostophycin. **(Figure S13, Table S20)**

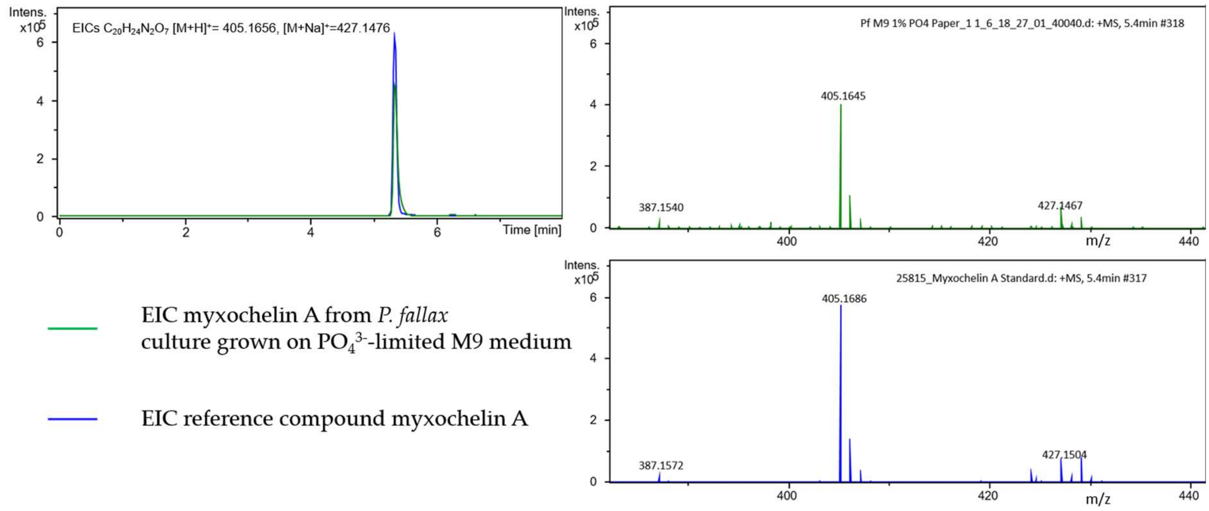

Figure S13. Comparison of chromatograms and MS-spectra of myxochelin A reference compound (blue) and EIC of sample *P. fallax* M9 1% PO_4^{3-} (green). Δppm = 10.1.

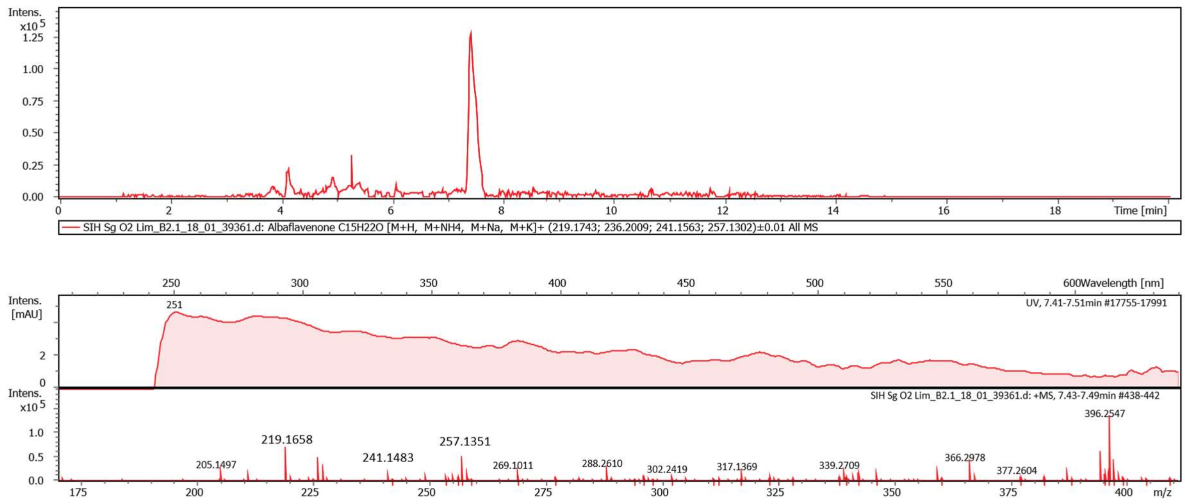

Figure S14. Extracted Ion Chromatogram and corresponding MS-spectrum of putative albaflavenone from oxygen limitation sample of *S. griseochromogenes* culture. The UV spectrum is shown above the MS spectrum. Δppm = 38.8.

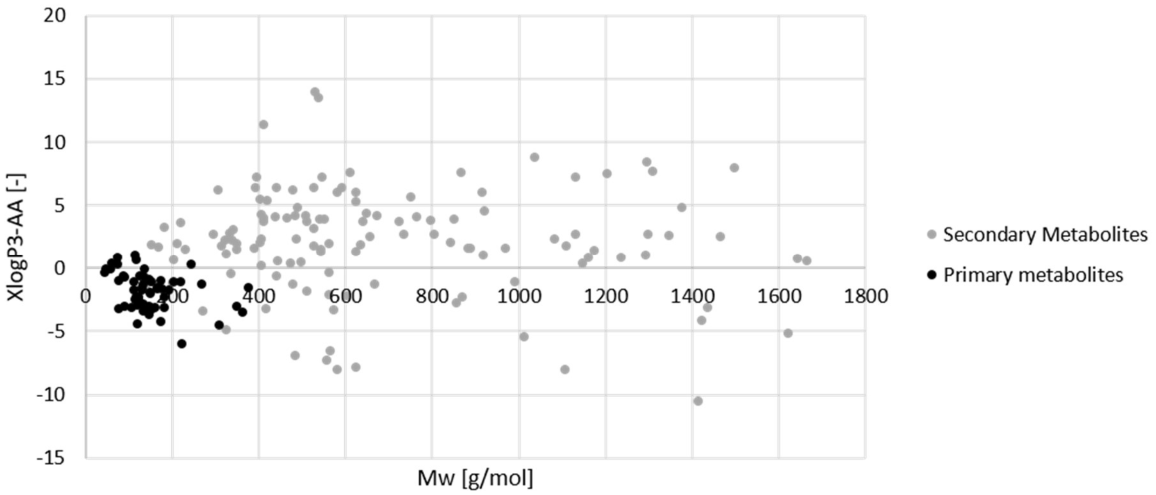

Figure S15. Comparison of physicochemical data of bacterial primary and secondary metabolites taken from PubChem.

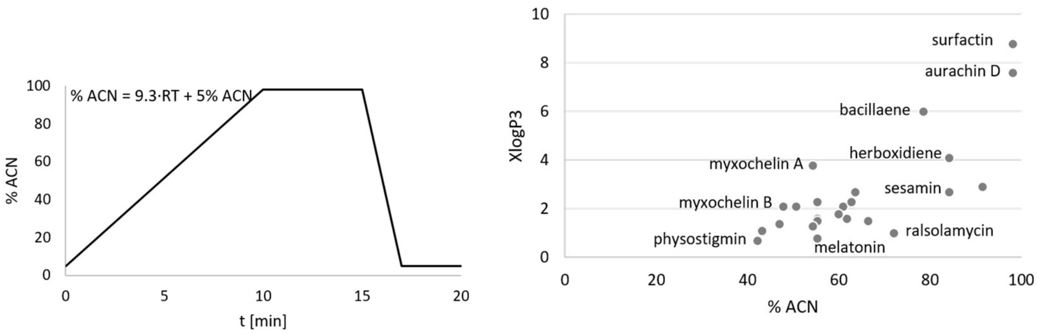

Figure S16. Visualization of employed HPLC method and correlation of logP values and ACN percentage at t_R.

Table S21. List of new mass features detected in extracts from *B. amyloliquefaciens* DSM7 including mass feature ID, $m/z_{measured}$, retention time (t_R), number of activating conditions and activating conditions.

ID	$m/z_{measured}$ [-]	t_R [min]	# of a.c.	activating conditions (a.c.)
**Ba1**	304.3004	8.51	1	Landy Medium
**Ba2**	332.3315	9.22	1	Landy Medium
**Ba3**	261.1232	4.5	4	GMS MgX, 40°C, LB Medium, Landy Medium
**Ba4**	338.1392	7.92	4	Landy Medium
**Ba5**	227.1389	3.92	3	GMS FeX, LB Medium, Landy Medium
**Ba6**	458.1596/ 436.172	5.9	1	Landy Medium
**Ba7**	277.1194	4.78	1	Landy Medium
**Ba8**	1057.57	7.21	1	LB Medium
**Ba9**	1071.5832	7.57	1	LB Medium
**Ba10**	1085.6	7.89	1	LB Medium
**Ba11**	250.1126	6.67	4	3% EtOH, 3% Tol, 40°C, LB Medium
**Ba12**	360.1934	4.45	1	LB Medium
**Ba13**	382.1563	6.64	2	GMS, LB Medium
**Ba14**	316.2487	7.7	2	+ Sg sup sf, + Pf sup auto
**Ba18**	418.2748	6.44	1	SP Medium
**Ba19**	346.2602	7.35	3	3% ACN, 25°C, + Sg sup sf
**Ba20**	510.3197	8.78	1	25°C
**Ba21**	233.1316	6.1	1	25°C
**Ba22**	363.1955	6.1	1	25°C
**Ba23**	228.223	10.33	1	40°C
**Ba24**	243.0854	6.13	1	40°C
**Ba25**	211.1408	6.13	1	40°C
**Ba27**	227.1728	5.79	3	3% EtOH, 3% Tol, 40°C
**Ba28**	311.1368	5.21	1	40°C
**Ba29**	225.041	6.69	1	40°C
**Ba30**	213.1574	5.3	2	3% EtOH, 40°C
**Ba31**	453.2702	4.09	1	40°C
**Ba32**	227.1529	5.41	1	GMS
**Ba35**	398.2633/ 382.2858	10.27	3	GMS MgX, GMS FeX, GMS
**Ba36**	495.2603	9.81	5	3% EtOH, 3% Tol, GMS MgX, GMS FeX, GMS
**Ba38**	370.2349	5.97	2	GMS FeX, GMS
**Ba39**	537.1458	4.77	2	GMS FeX, GMS
**Ba41**	412.2427/ 426.2224	7.16	1	GMS
**Ba42**	480.2127	7.41	1	GMS
**Ba43**	433.2961	10.43	5	3% EtOH, 3% Tol, GMS MgX, GMS FeX, GMS
**Ba44**	390.1644	7.43	1	GMS
**Ba46**	298.2746	9.94	1	GMS
**Ba48**	296.2594	9.49	1	GMS

**Ba52**	623.3655	8.36	1	GMS
**Ba53**	328.1826	9.81	2	GMS FeX, GMS
**Ba54**	263.2369	9.05	1	GMS
**Ba55**	317.2691	8.36	1	GMS
**Ba56**	607.1897	5.21	1	GMS FeX
**Ba57**	265.1273	3.74	1	GMS FeX
**Ba58**	883.2646	6.07	1	GMS FeX
**Ba60**	708.2496	4.85	1	GMS FeX
**Ba61**	218.1372	4.51	1	GMS FeX
**Ba62**	384.2504	6.4	1	GMS FeX
**Ba63**	901.275	5.85	1	GMS FeX
**Ba64**	225.1371	10.96	1	GMS FeX
**Ba65**	287.1968	5.43	1	M9 1% PO_4^{3-}
**Ba68**	341.1802	8.8	2	M9 Medium, + Cc Pellet
**Ba69**	359.1908	7.14	1	M9 Medium
**Ba70**	401.2043	8.1	1	M9 Medium
**Ba71**	354.4145	9.85	1	M9 Medium
**Ba72**	325.2259	6.91	1	3% EtOH
**Ba73**	240.1379	8.8	2	3% EtOH, 3% Tol
**Ba74**	269.2005	5.88	2	3% EtOH, 3% Tol
**Ba75**	343.2944	7.19	2	3% EtOH, 3% Tol
**Ba77**	250.118	9.36	2	3% EtOH, 3% Tol
**Ba78**	284.1386	6.02	2	3% EtOH, 3% Tol
**Ba79**	249.2057	8.48	2	3% EtOH, 3% Tol
**Ba80**	285.1047	6.25	2	3% EtOH, 3% Tol
**Ba81**	234.0579	8.31	2	3% EtOH, 3% Tol
**Ba82**	252.1074	9.47	1	3% EtOH
**Ba85**	296.2571	10.18	1	3% EtOH
**Ba86**	292.1067	5.88	2	3% EtOH, 3% Tol
**Ba87**	987.1246	8.33	1	3% EtOH
**Ba88**	328.2837	6.77	2	3% EtOH, 3% Tol
**Ba89**	443.2566	4.99	2	+ Sg sup sf, + Sg sup auto
**Ba90**	625.3203	4.21	1	+ Sg sup sf
**Ba91**	243.1407	4.19	1	+ Sg sup sf
**Ba92**	654.2732	4.31	1	+ Sg sup sf
**Ba93**	401.2462	5.07	1	+ Sg sup sf
**Ba94**	496.1674	4.04	1	+ Sg sup sf
**Ba95**	565.1014	10.79	5	+ Rj sup sf, + Cc sup sf, + Pf sup auto, + Pf Pellet
**Ba96**	553.3055	4.5	1	+ Rj sup sf
**Ba97**	396.9055	6.32	2	+ Rj sup sf, + Pf Pellet
**Ba98**	252.0972	10.79	2	+ Rj sup sf, + Pf Pellet
**Ba99**	279.1207	5.44	1	+ Rj sup sf
**Ba100**	727.5254	8.53	1	+ Rj sup sf
**Ba101**	256.2317	5.91	1	+ Cc sup sf
**Ba102**	274.2421	5.91	1	+ Cc sup sf

**Ba103**	268.1930	5.54	1	+ Cc sup sf
**Ba104**	743.411	7.3	1	+ Cc sup sf
**Ba105**	370.198	7.23	2	+ Cc sup sf, + Pf sup auto
**Ba106**	254.1079	4.76	1	+ Rj sup auto
**Ba107**	440.2823	8.46	1	+ Pf sup auto
**Ba108**	553.3378	4.36	1	+ Pf sup auto
**Ba109**	385.2492	4.28	1	+ Pf sup auto
**Ba110**	439.1387	9.25	1	+ Pf sup auto
**Ba111**	424.134	9.3	1	3% DMSO
**Ba112**	279.0808	5.04	1	3% DMSO
**Ba113**	257.1294	7.62	1	3% ACN
**Ba114**	226.1469	8.95	1	3% ACN
**Ba115**	376.1669	8.71	1	3% ACN
**Ba116**	415.2023	9.28	1	3% ACN
**Ba117**	297.1603	8.32	1	3% ACN
**Ba118**	399.2079	8.95	1	3% ACN
**Ba119**	269.1392	8.19	1	3% ACN
**Ba120**	301.1651	8.19	1	3% ACN
**Ba121**	273.1487	9.37	1	3% ACN
**Ba122**	283.1449	8.42	1	3% ACN
**Ba123**	222.1129	5.49	1	3% ACN
**Ba124**	230.0814	5.29	1	3% ACN
**Ba125**	241.1227	9.37	1	3% ACN
**Ba126**	529.2658	6.37	1	3% ACN
**Ba127**	310.1668	5.9	1	3% ACN
**Ba128**	260.1863	7.21	1	3% ACN
**Ba129**	360.275	7.51	1	3% ACN
**Ba130**	255.1499	7.95	2	3% ACN, + Cc Pellet
**Ba131**	305.1752	9.37	1	3% ACN
**Ba132**	274.202	7.5	1	3% ACN
**Ba133**	310.1408	6.1	1	3% ACN
**Ba134**	340.1774	6.15	1	3% ACN
**Ba135**	233.1257	8.24	1	3% ACN
**Ba136**	463.286	7.9	1	3% ACN
**Ba137**	421.2402	7.9	1	3% ACN
**Ba138**	403.2243	8.78	1	3% ACN
**Ba139**	243.135	3.81	1	3% ACN
**Ba140**	236.1285	5.95	1	3% ACN
**Ba141**	357.1615	8.56	1	3% ACN
**Ba142**	269.2034	5.74	1	+ Cc Pellet
**Ba143**	279.106	9.16	1	+ Cc Pellet
**Ba144**	250.1234	8.15	1	+ Cc Pellet
**Ba145**	445.2617	8.96	1	+ Cc Pellet

Table S22. List of new mass features detected in extracts from *C. coralloides* DSM2259 including mass feature ID, $m/z_{measured}$, retention time (t_R), number of activating conditions and activating conditions.

ID	$m/z_{measured}$ [-]	t_R [min]	# of a.c.	activating conditions (a.c.)
**Cc1**	360.3602	9.4	1	M9 1% PO_4^{3-}
**Cc6**	385.1893	9.2	1	+ Sg sup auto
**Cc8**	793.3483	8.06	1	MD1 medium
**Cc11**	707.3117	7.7	1	MD1 medium
**Cc12**	332.3299	8.7	3	0,5% Tol, M9 medium, M9 1% PO_4^{3-}
**Cc18**	284.2593	7.7	1	MD1+G medium
**Cc21**	304.2975	8.0	5	M9 1% PO_4^{3-}, M9 medium, + Rj sup sf, + Pf sup sf, + Ba sup sf
**Cc22**	357.1579	10.2	2	0,5% Tol, GMS FeX
**Cc27**	349.2323	6.5	1	+ Ba P
**Cc29**	361.265	7.23	1	+ Ba sup sf
**Cc42**	601.3558	4.68	3	+ Sg sup sf, + Ba sup sf, + Rj sup sf
**Cc49**	343.2947	6.97	1	3% EtOH
**Cc50**	371.3278	7.82	1	3% EtOH
**Cc58**	348.322	6.79	1	+ Pf sup sf
**Cc59**	342.3742	7.46	2	+ Ba sup sf, + Pf sup sf
**Cc63**	587.3396	4.49	4	+ Rj sup auto, + Pf sup auto, + Ba sup auto, + Ba sup sf
**Cc84**	347.1532	6.18	1	+ Ba sup sf
**Cc90**	393.2525	3.13	1	+ Rj sup auto
**Cc102**	243.0859	5.48	1	40°C
**Cc104**	268.0811	5.26	1	40°C
**Cc105**	231.1141	5.95	1	40°C
**Cc146**	443.2531	4.6	1	+ Sg sup auto
**Cc147**	487.199	5.98	1	+ Sg sup auto
**Cc148**	399.172	9.3	1	+ Sg sup auto
**Cc149**	435.2194	6.52	1	+ Sg sup auto
**Cc154**	521.2413	3.01	1	+ Ba sup auto
**Cc155**	387.2056	5.51	1	+ Pf sup auto
**Cc167**	254.1018	5.14	1	40°C
**Cc181**	242.2838	7.82	2	GMS FeX, GMS MgX
**Cc183**	214.2525	7.13	3	GMS, GMS FeX, GMS MgX
**Cc186**	371.1742	5.59	1	GMS FeX
**Cc187**	359.1737	5.83	1	GMS FeX
**Cc189**	375.1688	6.22	1	GMS FeX
**Cc190**	284.1579	6.45	1	+ Pf sup sf
**Cc193**	359.1737	8.33	1	GMS FeX
**Cc210**	566.2988	9.64	1	GMS FeX
**Cc243**	380.2758	6.67	1	M9 FeX
**Cc244**	330.2638	5.81	1	M9 FeX

**Cc249**	340.2847	6.67	3	M9 FeX, + Rj sup sf, + Pf sup sf
**Cc250**	293.2003	6.64	1	M9 FeX
**Cc274**	585.3623	4.82	1	O2-Lim
**Cc275**	373.1203	7.18	1	O2-Lim
**Cc276**	337.1464	10.57	1	O2-Lim

Table S23. List of new mass features detected in extracts from *P. fallax* HKI727 including mass feature ID, $m/z_{measured}$, retention time (t_R), number of activating conditions and activating conditions.

ID	$m/z_{measured}$ [-]	t_R [min]	# of a.c.	activating conditions (a.c.)
**Pf2**	312.2872	7.92	1	NB medium
**Pf12**	441.1847	1.19	1	NB medium
**Pf17**	300.324	8.78	1	NB medium
**Pf22**	276.1065	1.12	1	NB medium
**Pf24**	330.2622	8.66	1	SP medium
**Pf27**	521.2367	2.74	1	TSB medium
**Pf31**	247.1279	1.8	1	1% DMSO @35°C
**Pf43**	313.2007	6.17	1	1% EtOH @25°C
**Pf48**	314.3031	7.99	2	+ Ba Pellet, NB medium
**Pf54**	353.2053	5.49	1	SP medium
**Pf55**	433.1931	6.18	2	SP medium, + Cc sup auto
**Pf56**	356.1453	1.85	1	1% DMSO @35°C
**Pf57**	340.3579	8.43	1	TSB medium
**Pf60**	325.1764	4.89	2	SP medium, + Cc sup sf
**Pf61**	342.3715	8.36	1	1% Tol
**Pf62**	312.3621	8.95	2	1% DMSO @35°C, SP medium
**Pf63**	272.1582	4.46	8	1% DMSO @35°C, 0,5% DMSO, SP medium, + Cc sup sf, + Ba sup sf, + Cc Pellet, + Ba Pellet, 35°C
**Pf69**	298.3445	8.63	5	1% DMSO @35°C, 1% Tol, 0,5% Tol @ 25°C, TSB medium, SP medium
**Pf77**	255.1692	4.72	1	TSB medium
**Pf92**	358.1384	4.24	3	1% DMSO @35°C, 3% EtOH, 25°C
**Pf93**	316.182	3.74	1	25°C
**Pf95**	383.2239	4.06	1	25°C
**Pf96**	343.1938	2.27	2	1% DMSO @35°C, 25°C
**Pf97**	295.1247	3.33	1	25°C
**Pf98**	250.9953	7.87	2	35°C, 25°C
**Pf99**	316.243	9.29	1	25°C
**Pf103**	451.1426	3.72	5	1% DMSO @35°C, 3% DMSO, 0,5% DMSO, 3% EtOH, 1% EtOH @25°C
**Pf104**	619.2468	4.82	2	0,5% DMSO, 1% EtOH @25°C
**Pf105**	375.1246	4.83	1	0,5% DMSO
**Pf106**	387.1464	4.55	7	1% DMSO @35°C, 3% DMSO, 0,5% DMSO, 3% EtOH, 1% EtOH @25°C, 1% Tol, 0,5% Tol @ 25°C

**Pf108**	716.4562	5.14	7	1% DMSO @35°C, 3% DMSO, 0,5% DMSO, 3% EtOH, 1% EtOH @25°C, 1% Tol, 0,5% Tol @ 25°C
**Pf109**	315.2194	6.3	6	0,5% DMSO, + Cc sup sf, + Ba sup sf, + Ba sup auto, + Cc Pellet, + Ba Pellet
**Pf110**	575.5	8.14	9	1% DMSO @35°C, 3% DMSO, 0,5% DMSO, 3% EtOH, 1% EtOH @25°C, 0,5% EtOH @ 35°C, 3% Tol @ 35°C, 1% Tol, 0,5% Tol @ 25°C
**Pf111**	705.4926	8.14	9	1% DMSO @35°C, 3% DMSO, 0,5% DMSO, 3% EtOH, 1% EtOH @25°C, 0,5% EtOH @ 35°C, 3% Tol @ 35°C, 1% Tol, 0,5% Tol @ 25°C
**Pf112**	900.6918	8.14	9	1% DMSO @35°C, 3% DMSO, 0,5% DMSO, 3% EtOH, 1% EtOH @25°C, 0,5% EtOH @ 35°C, 3% Tol @ 35°C, 1% Tol, 0,5% Tol @ 25°C
**Pf114**	272.2238	9.64	9	1% DMSO @35°C, 3% DMSO, 0,5% DMSO, 3% EtOH, 1% EtOH @25°C, 0,5% EtOH @ 35°C, 3% Tol @ 35°C, 1% Tol, 0,5% Tol @ 25°C
**Pf115**	406.106	1.,76	1	0,5% DMSO
**Pf120**	586.1848	3.91	5	1% DMSO @35°C, 3% DMSO, 3% EtOH, 1% EtOH @25°C, 0,5% Tol @ 25°C
**Pf122**	351.1488	3.38	3	3% EtOH, 0,5% Tol @ 25°C
**Pf123**	723.2384	3.91	5	1% DMSO @35°C, 3% DMSO, 3% EtOH, 1% EtOH @25°C, 0,5% Tol @ 25°C
**Pf124**	243.1353	3.97	1	1% DMSO @35°C
**Pf125**	1015.4444	4.82	6	1% DMSO @35°C, 3% DMSO, 3% EtOH, 1% EtOH @25°C, 1% Tol, 0,5% Tol @ 25°C
**Pf129**	532.2226	10.71	3	3% DMSO, 3% EtOH, 1% EtOH @25°C
**Pf131**	321.1285	5.27	1	3% DMSO
**Pf132**	292.1819	5.46	2	3% DMSO, 1% EtOH @25°C
**Pf134**	421.2044	10.42	1	3% DMSO
**Pf144**	332.176	10.3	1	3% EtOH
**Pf159**	270.1444	5.07	3	3% DMSO, 3% EtOH, 1% EtOH @25°C
**Pf160**	421.1298	4.63	1	1% EtOH @25°C
**Pf161**	505.2129	5.38	1	3% EtOH
**Pf163**	270.1459	5.02	3	3% EtOH, 1% Tol, 0,5% Tol @ 25°C
**Pf164**	383.2291	3.72	1	3% EtOH
**Pf165**	523.2568	4.82	2	3% EtOH, 0,5% Tol @ 25°C
**Pf167**	1258.5694	4.8	1	0,5% Tol @ 25°C
**Pf169**	1187.9476	8.14	3	3% DMSO, 3% EtOH, 1% EtOH @25°C
**Pf171**	342.1983	6.27	3	3% DMSO, GMS FeX, M9 1% PO_4^{3-}
**Pf175**	354.2189	6.37	1	1% EtOH @25°C
**Pf176**	415.2124	8.11	1	3% EtOH
**Pf178**	356.1772	6.85	1	3% EtOH
**Pf184**	274.0961	9.22	1	3% DMSO
**Pf185**	245.1278	9.31	3	3% DMSO, 3% EtOH, 1% EtOH @25°C
**Pf186**	799.178	9.36	2	3% EtOH, 1% EtOH @25°C

**Pf192**	229.1219	2.22	1	1% DMSO @35°C
**Pf201**	243.1963	8.07	1	3% EtOH
**Pf202**	244.2295	8.33	2	1% DMSO @35°C, 3% EtOH
**Pf204**	456.264	10.74	1	3% EtOH
**Pf205**	299.2589	10.47	1	3% EtOH
**Pf208**	357.1772	10.28	1	3% EtOH
**Pf213**	393.2579	4.48	2	3% EtOH, 0,5% Tol @ 25°C
**Pf214**	475.2567	4.09	1	3% EtOH
**Pf217**	314.2695	8.38	1	3% EtOH
**Pf218**	378.2036	3.45	1	3% EtOH
**Pf220**	225.149	8.93	1	3% EtOH
**Pf221**	328.2241	3.99	1	3% EtOH
**Pf222**	298.2747	9.68	1	3% EtOH
**Pf223**	320.1758	5.95	1	3% EtOH
**Pf224**	338.2677	7.87	1	3% EtOH
**Pf228**	342.2395	4.36	1	3% EtOH
**Pf231**	943.6278	5.37	1	3% EtOH
**Pf232**	440.2512	3.54	1	3% EtOH
**Pf240**	467.2943	4.43	1	0,5% Tol @ 25°C
**Pf245**	583.4444	10.42	2	1% DMSO @35°C, 0,5% Tol @ 25°C
**Pf258**	330.2033	1.98	1	1% DMSO @35°C
**Pf259**	333.1697	6.18	1	1% EtOH @25°C
**Pf260**	334.2294	7.01	1	1% EtOH @25°C
**Pf261**	242.0869	3.15	1	1% EtOH @25°C
**Pf279**	254.1054	5.09	1	+ Cc sup auto
**Pf280**	327.2079	5.41	1	+ Cc sup auto
**Pf282**	314.3063	8.07	3	+ Sg sup sf, + Ba sup sf, + Sg sup auto
**Pf285**	300.2911	7.82	1	+ Sg sup auto
**Pf286**	316.3225	8.31	4	+ Sg sup sf, + Ba sup sf, + Sg sup auto, + Ba Pellet
**Pf287**	316.3225	6.94	1	+ Sg sup auto
**Pf290**	290.1301	8.76	2	+ Sg sup auto, + Rj sup auto
**Pf292**	235.1094	1.33	1	+ Rj sup auto
**Pf293**	381.1715	9.42	1	+ Rj sup auto
**Pf294**	274.1636	10.33	3	+ Sg sup sf, + Cc sup sf, + Ba sup sf
**Pf295**	269.2061	5.75	1	+ Sg sup sf
**Pf297**	358.206	8.78	3	+ Sg sup sf, + Cc sup sf, + Ba sup sf
**Pf298**	243.1388	3.87	1	+ Sg sup sf
**Pf300**	445.2646	8.95	3	+ Sg sup sf, + Cc sup sf, + Ba sup sf
**Pf301**	387.1976	5.98	6	+ Sg sup sf, + Cc sup sf, + Ba sup sf, + Sg Pellet, + Cc Pellet, + Ba Pellet
**Pf304**	231.0816	4.46	2	+ Sg sup sf, + Cc sup sf
**Pf307**	298.1009	1.83	1	+ Sg sup sf
**Pf308**	418.2292	8.07	1	+ Sg sup sf
**Pf309**	255.1543	7.67	2	+ Sg sup sf, + Ba sup sf
**Pf310**	365.348	6.36	1	+ Ba sup sf

**Pf311**	263.1417	5.01	4	+ Cc sup sf, + Ba sup sf, + Cc Pellet, + Ba Pellet
**Pf314**	279.188	6.43	1	+ Cc sup sf
**Pf315**	296.1378	1.86	1	+ Cc sup sf
**Pf316**	496.1834	7.82	2	GMS FeX, M9 1% PO_4^{3-}
**Pf317**	331.2029	5.53	1	GMS FeX
**Pf318**	351.1692	5.61	1	GMS FeX
**Pf319**	339.1856	6.67	1	GMS MgX
**Pf321**	470.2255	5.83	1	M9 medium
**Pf322**	427.0937	5.26	1	M9 medium
**Pf323**	289.0578	4.87	2	M9 1% PO_4^{3-}, M9 medium
**Pf324**	348.2185	6.0	1	M9 medium
**Pf325**	459.0651	5.54	1	M9 medium
**Pf326**	311.1916	5.8	1	M9 medium
**Pf327**	659.139	3.67	1	M9 medium
**Pf328**	486.2024	6.18	2	M9 1% PO_4^{3-}, M9 medium
**Pf329**	491.1096	4.09	1	M9 medium
**Pf330**	691.1106	4.11	1	M9 medium
**Pf331**	509.1023	4.43	1	M9 medium
**Pf332**	428.2193	7.69	1	M9 1% PO_4^{3-}
**Pf333**	395.1362	6.39	1	M9 1% PO_4^{3-}
**Pf334**	435.1762	5.8	1	M9 1% PO_4^{3-}
**Pf335**	390.1625	7.35	1	M9 1% PO_4^{3-}
**Pf336**	405.1645	5.38	1	M9 1% PO_4^{3-}
**Pf337**	360.2178	6.1	1	M9 1% PO_4^{3-}
**Pf338**	227.1541	8.38	1	M9 1% PO_4^{3-}
**Pf339**	206.1022	4.97	1	M9 1% PO_4^{3-}
**Pf340**	448.2318	4.95	1	M9 1% PO_4^{3-}
**Pf342**	394.3477	8.38	1	M9 1% PO_4^{3-}
**Pf343**	398.1851	8.04	1	M9 1% PO_4^{3-}
**Pf344**	226.1465	6.0	1	M9 1% PO_4^{3-}
**Pf345**	336.0843	4.92	1	M9 1% PO_4^{3-}
**Pf347**	230.2108	6.2	1	M9 1% PO_4^{3-}
**Pf348**	387.1569	6.73	1	M9 1% PO_4^{3-}
**Pf350**	313.2025	6.42	1	+ Ba sup auto
**Pf351**	309.1455	5.63	1	+ Ba sup auto

Table S24. List of new mass features detected in extracts from *R. jostii* DSM44719 including mass feature ID, $m/z_{measured}$, retention time (t_R), number of activating conditions and activating conditions.

**ID**	$m/z_{measured}$ **[-]**	t_R **[min]**	**# of a.c.**	**activating conditions (a.c.)**
**Rj1**	271.1523	6.72	4	+ Sg sup auto, + Cc sup auto, + Sg Pellet, + Cc Pellet
**Rj4**	480.2788	4.26	1	+ Sg Pellet
**Rj6**	331.0206	5.04	1	+ Sg Pellet
**Rj9**	217.1016	4.75	4	LB Medium, + Pf sup auto, + Ba sup auto, + Cc Pellet

**Rj10**	220.1203	4.83	1	+ Ba sup auto
**Rj11**	254.1763	6.37	3	O_2-Lim., + Cc sup auto, + Cc Pellet
**Rj13**	250.1136	6.37	4	O_2-Lim., + Pf sup auto, + Ba sup auto, + Cc Pellet
**Rj15**	287.2589	9.57	1	GMS MgX
**Rj16**	269.2126	8.88	2	GMS, Landy Medium
**Rj18**	271.2283	9.34	1	Landy Medium
**Rj20**	257.2124	8.63	2	GMS, Landy Medium
**Rj22**	255.2332	8.76	3	GMS MgX, GMS, Landy Medium
**Rj24**	213.1855	7.18	4	GMS MgX, GMS FeX, GMS, Landy Medium
**Rj26**	227.2015	7.72	4	GMS MgX, GMS FeX, GMS, Landy Medium
**Rj31**	203.1395	3.84	1	+ Pf sup auto
**Rj45**	296.138	1.92	1	+ Pf Pellet
**Rj47**	319.2868	10,17	2	+ Pf Pellet, 23°C
**Rj65**	243.1308	3.91	1	+ Sg sup auto
**Rj88**	261.1225	4.08	1	NB Medium
**Rj89**	453.2682	3.79	1	NB Medium
**Rj90**	292.1063	5.71	1	NB Medium
**Rj91**	521.2382	4.03	1	NB Medium
**Rj92**	300.1362	3.96	1	NB Medium
**Rj93**	275.0838	4.28	1	NB Medium
**Rj94**	297.1359	4.94	1	NB Medium
**Rj95**	240.0687	6.17	1	O_2-Lim.
**Rj98**	237.2205	10.33	1	23°C
**Rj120**	285.1345	1.87	1	O_2-Lim.
**Rj141**	435.3022	8.1	1	M9 FeX
**Rj142**	414.2576	8.63	1	M9 1% PO_4^{3-}
**Rj144**	307.2184	7.01	1	M9 FeX
**Rj146**	354.2854	8.58	1	M9 1% PO_4^{3-}
**Rj165**	230.2121	6.23	1	M9 FeX, M9 1% PO_4^{3-}
**Rj166**	216.1963	5.75	1	M9 FeX
**Rj185**	240.233	7.16	1	Landy Medium
**Rj214**	342.235	4.53	1	0,5% EtOH
**Rj215**	328.218	4.26	1	0,5% EtOH
**Rj217**	318.1992	3.84	1	0,5% EtOH
**Rj239**	430.1826	10.4	1	Landy Medium
**Rj240**	250.1454	4.1	1	Landy Medium
**Rj241**	228.1038	8.07	1	Landy Medium
**Rj242**	303.2547	7.92	3	GMS MgX, GMS FeX, Landy Medium
**Rj243**	271.2279	7.45	2	GMS MgX, Landy Medium
**Rj245**	354.1506	10.18	1	Landy Medium
**Rj246**	456.1609	6.81	1	Landy Medium
**Rj247**	370.1819	9.25	1	Landy Medium
**Rj248**	393.1829	5.83	1	Landy Medium
**Rj249**	264.1609	4.94	1	Landy Medium
**Rj250**	475.1665	8.87	1	Landy Medium

**Rj251**	306.1502	8.88	1	Landy Medium
**Rj252**	287.2237	8.04	2	GMS FeX, Landy Medium
**Rj253**	267.1954	7.81	4	GMS MgX, GMS FeX, GMS, Landy Medium
**Rj254**	503.1978	9.09	1	Landy Medium
**Rj255**	254.2478	7.87	2	GMS FeX, Landy Medium
**Rj256**	463.2859	7.89	2	GMS FeX, GMS
**Rj257**	271.1878	7.33	3	GMS MgX, GMS FeX, GMS
**Rj258**	533.4829	10.81	2	GMS FeX, GMS
**Rj259**	239.1619	6.84	2	GMS FeX, GMS
**Rj260**	321.1475	4.46	2	GMS FeX, GMS
**Rj261**	281.2092	8.34	3	GMS MgX, GMS FeX, GMS
**Rj262**	293.2093	8.43	2	GMS FeX, GMS
**Rj263**	270.2415	7.53	2	GMS FeX, GMS
**Rj264**	263.158	10.49	2	GMS FeX, GMS
**Rj265**	241.265	8.34	1	GMS
**Rj266**	325.235	7.94	1	GMS
**Rj267**	245.0939	5.22	2	GMS FeX, GMS
**Rj269**	253.1411	5.48	2	GMS FeX, GMS
**Rj270**	235.2048	7.53	2	GMS FeX, GMS
**Rj271**	284.2574	7.99	2	GMS FeX, GMS
**Rj272**	253.2166	8.43	3	GMS MgX, GMS FeX, GMS
**Rj273**	267.1574	5.81	2	GMS FeX, GMS
**Rj275**	307.2246	8.92	1	GMS
**Rj276**	229.143	5.81	1	GMS
**Rj277**	275.2206	7.03	2	GMS FeX, GMS
**Rj278**	456.31	10.54	1	GMS
**Rj279**	393.2475	3.27	2	GMS FeX, GMS
**Rj280**	244.2266	6.96	2	GMS FeX, GMS
**Rj281**	311.2192	7.48	2	GMS MgX, GMS
**Rj282**	247.19	6.15	2	GMS FeX, GMS
**Rj284**	482.3627	8.88	1	GMS
**Rj285**	257.2117	6.47	1	GMS FeX
**Rj286**	265.1777	7.33	1	GMS FeX
**Rj287**	257.2111	7.03	1	GMS FeX
**Rj288**	241.2165	8.33	2	GMS MgX, GMS FeX
**Rj289**	226.2165	6.96	1	GMS FeX
**Rj290**	428.2183	8.06	1	GMS FeX
**Rj291**	279.1933	7.85	1	GMS FeX
**Rj292**	479.1797	10.59	1	GMS FeX
**Rj293**	295.1878	7.55	1	GMS FeX
**Rj294**	274.2367	6.29	1	GMS FeX
**Rj295**	319.2428	6.71	1	GMS FeX
**Rj296**	256.227	7.74	1	GMS FeX
**Rj297**	301.2369	7.04	1	GMS FeX
**Rj298**	272.258	7.89	1	GMS FeX

**Rj299**	275.2275	8.02	1	GMS FeX
**Rj300**	307.1883	7.26	1	GMS FeX
**Rj301**	275.2212	6.47	1	GMS FeX
**Rj302**	259.1918	7.06	1	GMS FeX
**Rj303**	245.0419	4.31	1	GMS FeX
**Rj304**	243.1948	6.05	1	GMS FeX
**Rj305**	256.2543	5.61	1	GMS FeX
**Rj306**	511.2551	6.37	1	GMS FeX
**Rj307**	325.2851	6.89	1	GMS FeX
**Rj308**	243.1951	6.57	1	GMS FeX
**Rj309**	233.1723	5.75	1	GMS FeX
**Rj310**	269.211	7.18	1	GMS FeX
**Rj311**	255.1949	8.26	1	GMS FeX
**Rj312**	291.1935	8.11	1	GMS FeX
**Rj313**	210.0218	4.19	1	GMS FeX
**Rj314**	375.1819	8.09	1	+ Sg sup sf
**Rj315**	243.1338	4.06	1	+ Sg sup sf
**Rj316**	443.2487	4.9	1	+ Sg sup sf
**Rj317**	227.1387	3.76	1	+ Sg sup sf
**Rj318**	507.2416	4.06	1	+ Sg sup sf
**Rj319**	255.2306	9.84	1	+ Sg sup sf
**Rj320**	385.1935	9.22	1	GMS MgX
**Rj321**	363.1419	7.06	1	GMS MgX
**Rj322**	277.1001	5.27	1	GMS MgX
**Rj323**	226.1489	8.93	1	GMS MgX
**Rj324**	348.1733	6.56	1	GMS MgX
**Rj325**	326.1915	6.59	1	GMS MgX
**Rj326**	329.1726	4.82	1	GMS MgX
**Rj327**	399.2094	8.93	1	GMS MgX
**Rj328**	281.0945	6.1	1	GMS MgX
**Rj329**	398.1896	8.26	1	GMS MgX
**Rj330**	381.183	7.96	1	GMS MgX
**Rj331**	262.11	5.33	1	GMS MgX
**Rj332**	243.1518	8.28	1	GMS MgX
**Rj333**	227.203	8.12	1	GMS MgX
**Rj334**	258.11541	5.87	1	GMS MgX
**Rj336**	298.2339	7.33	1	GMS MgX
**Rj337**	415.2042	9.25	1	GMS MgX
**Rj338**	255.0788	5.92	1	GMS MgX
**Rj339**	400.2045	6.79	1	GMS MgX
**Rj340**	259.229	8.63	1	GMS MgX
**Rj341**	334.2575	5.55	1	GMS MgX
**Rj342**	222.115	568	1	GMS MgX
**Rj343**	224.1293	6.35	1	GMS MgX

Table S25. List of new mass features detected in extracts from *S. griseochromogenes* DSM40499 including mass feature ID, $m/z_{measured}$, retention time (t_R), number of activating conditions and activating conditions.

ID	m/z measured [-]	t_R [min]	# of a.c.	activating conditions (a.c.)
**Sg1**	252.1864	4.06	1	TSB Medium
**Sg3**	236.1812	5.09	1	TSB Medium
**Sg4**	231.0645	7.04	1	TSB Medium
**Sg9**	998.5606	7.99	3	3% DMSO, 1% DMSO, CYH-Medium
**Sg11**	639.3028	5.09	1	CYH-Medium
**Sg12**	423.2058	8.36	1	CYH-Medium
**Sg13**	444.1734	3.84	1	CYH-Medium
**Sg14**	393.1806	6.56	1	Landy Medium
**Sg16**	425.1706	6.03	1	Landy Medium
**Sg18**	333.1586	6.56	1	Landy Medium
**Sg19**	393.1806	5.93	1	Landy Medium
**Sg21**	266.1162	6.4	1	Landy Medium
**Sg23**	305.2212	6.37	1	Landy Medium
**Sg30**	307.1419	6.88	1	Landy Medium
**Sg38**	259.1777	8.78	1	40°C
**Sg39**	208.0583	4.43	2	1% DMSO, 40°C
**Sg42**	209.042	5.34	1	40°C
**Sg43**	351.1784	6.44	1	40°C
**Sg44**	234.121	3.84	1	40°C
**Sg45**	414.1396	4.53	1	40°C
**Sg47**	447.0908	5.19	2	1% DMSO, 40°C
**Sg53**	365.1722	7.31	1	40°C
**Sg54**	235.1047	4.7	1	40°C
**Sg68**	325.2347	5.73	3	3% EtOH, 1% EtOH, SP-Medium
**Sg71**	269.066	4.83	1	3% EtOH
**Sg72**	297.0606	5.73	1	3% EtOH
**Sg73**	410.1465	7.07	1	3% EtOH
**Sg74**	532.2989	7.19	2	3% EtOH, 3% ACN
**Sg77**	301.217	5.95	2	3% EtOH, O2-Lim.
**Sg84**	359.1568	6.6	3	GMS FeX, GMS Medium, O2-Lim.
**Sg85**	386.3099	6.3	1	O2-Lim.
**Sg87**	420.2615	4.99	1	O2-Lim.
**Sg89**	379.184	5.02	1	GMS Medium
**Sg90**	362.2402	4.85	1	GMS Medium
**Sg91**	325.2652	5.04	1	GMS Medium
**Sg92**	378.1779	5.04	1	GMS Medium
**Sg94**	339.2226	10.12	1	GMS Medium
**Sg96**	337.2226	10.12	1	GMS FeX

**Sg97**	336.1746	4.75	1	GMS FeX	
**Sg101**	290.2841	7.87	1	GMS FeX	
**Sg103**	256.1512	8.53	1	GMS FeX	
**Sg104**	312.37	8.64	1	GMS FeX	
**Sg106**	418.3013	10.12	1	GMS FeX	
**Sg107**	369.2497	10.12	1	GMS FeX	
**Sg109**	353.219	9.69	1	GMS FeX	
**Sg115**	379.2431	6.49	2	M9 FeX, M9 1% PO_4^{3-}	
**Sg117**	587.3468	4.53	1	M9 FeX	
**Sg118**	295.1597	4.89	2	M9 FeX, M9 Medium	Cyclo-(Phe-Phe)
**Sg119**	583.3485	4.36	1	M9 FeX	
**Sg120**	823.4818	5.46	1	M9 FeX	
**Sg121**	243.1311	4.26	1	SP-Medium	
**Sg130**	585.3593	4.94	2	3% DMSO, 1% DMSO	
**Sg134**	342.3789	8.44	1	M9 1% PO_4^{3-}	
**Sg135**	430.1457	7.0	1	M9 FeX	
**Sg143**	340.3619	8.17	1	M9 1% PO_4^{3-}	
**Sg145**	257.109	5.61	1	0,5% ACN	
**Sg146**	208.0661	4.04	1	M9 1% PO_4^{3-}	
**Sg147**	348.2248	6.05	1	M9 1% PO_4^{3-}	
**Sg148**	428.2258	7.68	1	M9 1% PO_4^{3-}	
**Sg149**	317.1929	6.48	1	M9 1% PO_4^{3-}	
**Sg150**	395.1427	6.39	1	M9 1% PO_4^{3-}	
**Sg151**	327.1753	6.39	1	M9 1% PO_4^{3-}	
**Sg152**	515.4181	9.61	1	M9 1% PO_4^{3-}	
**Sg153**	259.1877	8.32	1	M9 1% PO_4^{3-}	
**Sg154**	390.1706	7.36	1	M9 1% PO_4^{3-}	
**Sg155**	345.225	8.27	1	M9 1% PO_4^{3-}	
**Sg156**	217.1394	6.26	1	M9 1% PO_4^{3-}	
**Sg157**	289.0644	4.94	1	M9 1% PO_4^{3-}	
**Sg158**	269.1721	9.0	1	M9 1% PO_4^{3-}	
**Sg159**	226.1524	6.05	1	M9 1% PO_4^{3-}	
**Sg160**	388.1754	5.78	1	M9 1% PO_4^{3-}	
**Sg161**	415.1217	4.04	1	M9 1% PO_4^{3-}	
**Sg163**	275.1451	4.33	1	M9 1% PO_4^{3-}	
**Sg164**	415.1316	4.33	1	M9 1% PO_4^{3-}	
**Sg165**	303.1781	6.63	1	M9 1% PO_4^{3-}	
**Sg167**	386.1801	6.71	1	M9 1% PO_4^{3-}	
**Sg168**	499.2702	6.51	1	M9 1% PO_4^{3-}	
**Sg172**	464.2239	6.12	1	M9 1% PO_4^{3-}	
**Sg175**	269.1178	5.24	1	M9 1% PO_4^{3-}	
**Sg176**	386.1798	6.75	1	M9 1% PO_4^{3-}	
**Sg181**	331.2088	7.78	1	M9 1% PO_4^{3-}	
**Sg184**	415.1232	4.11	1	M9 1% PO_4^{3-}	
**Sg185**	311.1457	4.84	1	M9 1% PO_4^{3-}	Cyclo-(Phe-Tyr)

**Sg187**	573.3914	5.53	1	M9 1% PO_4^{3-}
**Sg198**	573.3304	4.45	1	M9 FeX
**Sg199**	599.345	4.87	2	M9 FeX, + Pf sup auto
**Sg201**	393.2592	7.15	1	M9 FeX
**Sg203**	421.2689	7.8	1	M9 FeX
**Sg206**	471.2689	10.3	1	M9 FeX
**Sg207**	276.0933	5.11	1	M9 FeX
**Sg214**	363.2865	8.56	1	M9 FeX
**Sg217**	328.1495	7.65	1	GMS MgX
**Sg220**	220.1151	1.26	1	1% DMSO
**Sg221**	458.3749	10.03	1	1% DMSO
**Sg222**	532.2887	7.59	1	1% DMSO
**Sg224**	225.1171	5.22	1	1% DMSO
**Sg227**	584.3468	4.94	1	1% DMSO
**Sg228**	273.177	5.34	1	1% DMSO
**Sg242**	320.252	9.96	1	3% DMSO
**Sg251**	243.1386	3.86	2	0,5% ACN, + Rj sup sf
**Sg252**	836.6292	10.13	2	+ Ba Pellet
**Sg253**	354.3058	9.86	1	0,5% ACN
**Sg255**	266.1126	4.78	3	0,5% ACN, + Rj sup sf, + Ba sup sf
**Sg257**	457.4401	7.04	1	+ Ba Pellet
**Sg258**	431.4251	6.86	1	+ Ba Pellet
**Sg264**	425.2848	5.26	1	3% ACN
**Sg272**	240.2293	7.57	1	3% Tol
**Sg273**	230.2091	6.59	1	3% Tol
**Sg274**	244.2246	7.08	1	3% Tol
**Sg275**	272.2555	8.34	1	3% Tol
**Sg276**	267.2289	9.09	1	3% Tol
**Sg277**	216.1934	6.1	1	3% Tol
**Sg278**	255.2291	9.0	1	3% Tol
**Sg279**	270.2399	6.79	1	3% Tol
**Sg280**	264.2292	6.44	1	3% Tol
**Sg285**	284.2557	8.16	1	3% Tol
**Sg286**	270.2403	6.22	1	3% Tol
**Sg287**	264.2299	7.74	1	3% Tol
**Sg288**	228.1931	5.53	1	3% Tol
**Sg290**	271.2241	8.8	1	3% Tol
**Sg291**	284.2196	7.04	1	3% Tol
**Sg293**	266.2409/<br>282.2395	6.5	1	3% Tol
**Sg294**	253.2133	8.53	1	3% Tol
**Sg296**	301.2348	8.33	1	3% Tol
**Sg297**	300.2499	6.69	1	3% Tol
**Sg299**	284.2197	6.3	1	3% Tol
**Sg302**	252.2294	7.69	1	3% Tol

**Sg308**	271.2352	6.72	1	3% Tol
**Sg309**	266.2092	7.84	1	3% Tol
**Sg310**	254.2449	8.06	1	3% Tol
**Sg312**	300.2512	7.38	1	3% Tol
**Sg335**	1030.578	7.15	1	+ Pf Pellet
**Sg336**	279.138	3.28	6	+ Pf sup auto, + Cc sup auto, + Rj Pellet, + Pf Pellet, + Ba Pellet, + Cc Pellet
**Sg337**	219.1166	4.04	2	+ Rj Pellet, + Pf Pellet
**Sg340**	1040.741	9.93	1	+ Ba Pellet
**Sg341**	597.3904	5.6	3	+ Rj Pellet, + Ba Pellet, + Cc Pellet
**Sg342**	414.2611	8.31	1	+ Ba Pellet
**Sg345**	252.1871	3.73	2	+ Ba sup sf, + Rj Pellet
**Sg346**	401.343	3.73	1	+ Rj Pellet
**Sg352**	434,.3177	5.12	1	+ Rj sup sf
**Sg353**	420.3018	4.78	1	+ Rj sup sf
**Sg354**	290.1655	4.78	1	+ Rj sup sf
**Sg357**	763.4162	4.63	1	+ Cc sup sf
**Sg358**	545.3635	4.2	1	+ Ba sup auto
**Sg359**	487.3471	6.78	1	+ Ba sup auto
**Sg363**	205.0963	3.18	1	+ Pf sup auto
**Sg364**	615.3323	5.77	1	+ Pf sup auto
**Sg366**	229.0996	3.86	1	+ Rj sup auto
**Sg367**	258.132	5.82	1	+ Rj sup auto
**Sg368**	201.1228	2.25	1	+ Rj sup auto

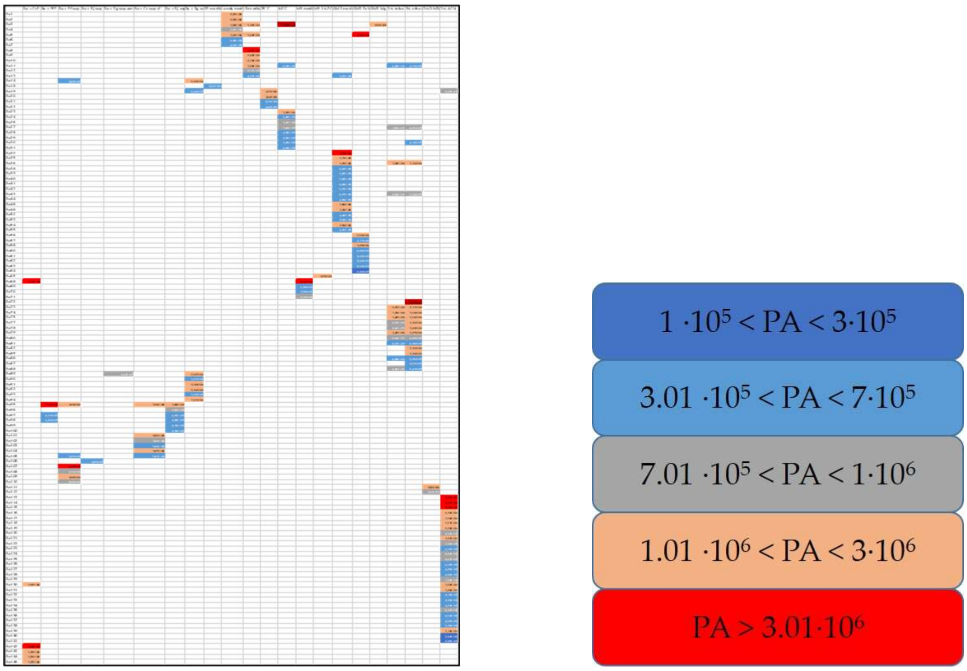

Figure S17. Heat map for new mass features of *B. amyloliquefaciens* DSM7 and utilized color code. PA = peak area. Rows = mass features, columns = producing conditions. Conditions which did not provoke any new mass features are not shown in the heat map. See separate excel file for detailed heat map.

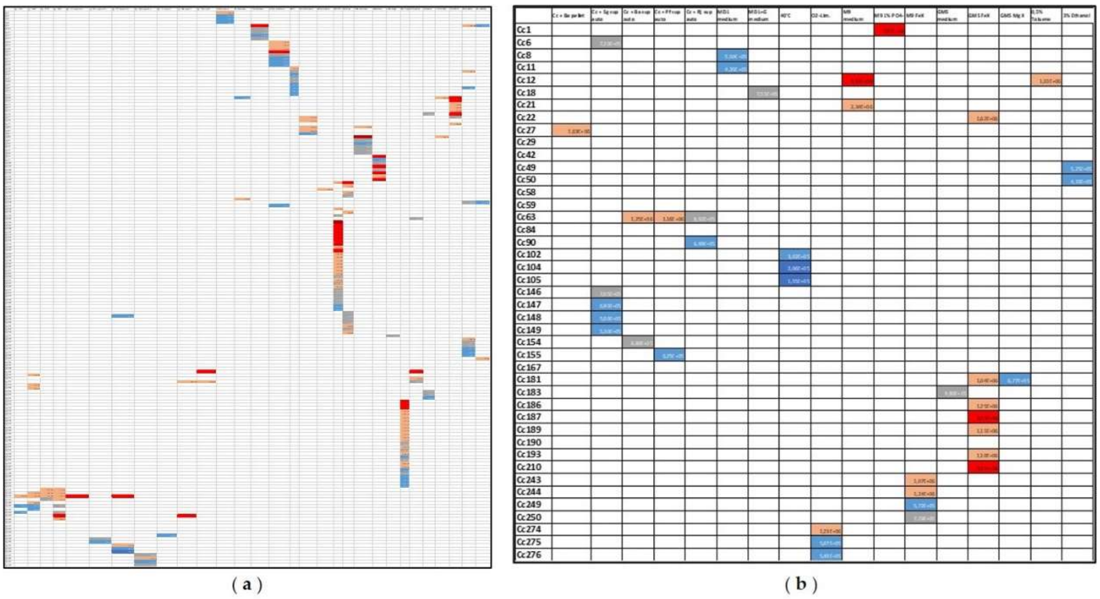

Figure 18. (**a**) Heat map of *S. griseochromogenes* DSM40499; (**b**) Heat map of *C. coralloides* DSM2259. Rows = mass features, columns = producing conditions. Conditions which did not provoke any new mass features are not shown in the heat map. See separate excel file for detailed heat map.

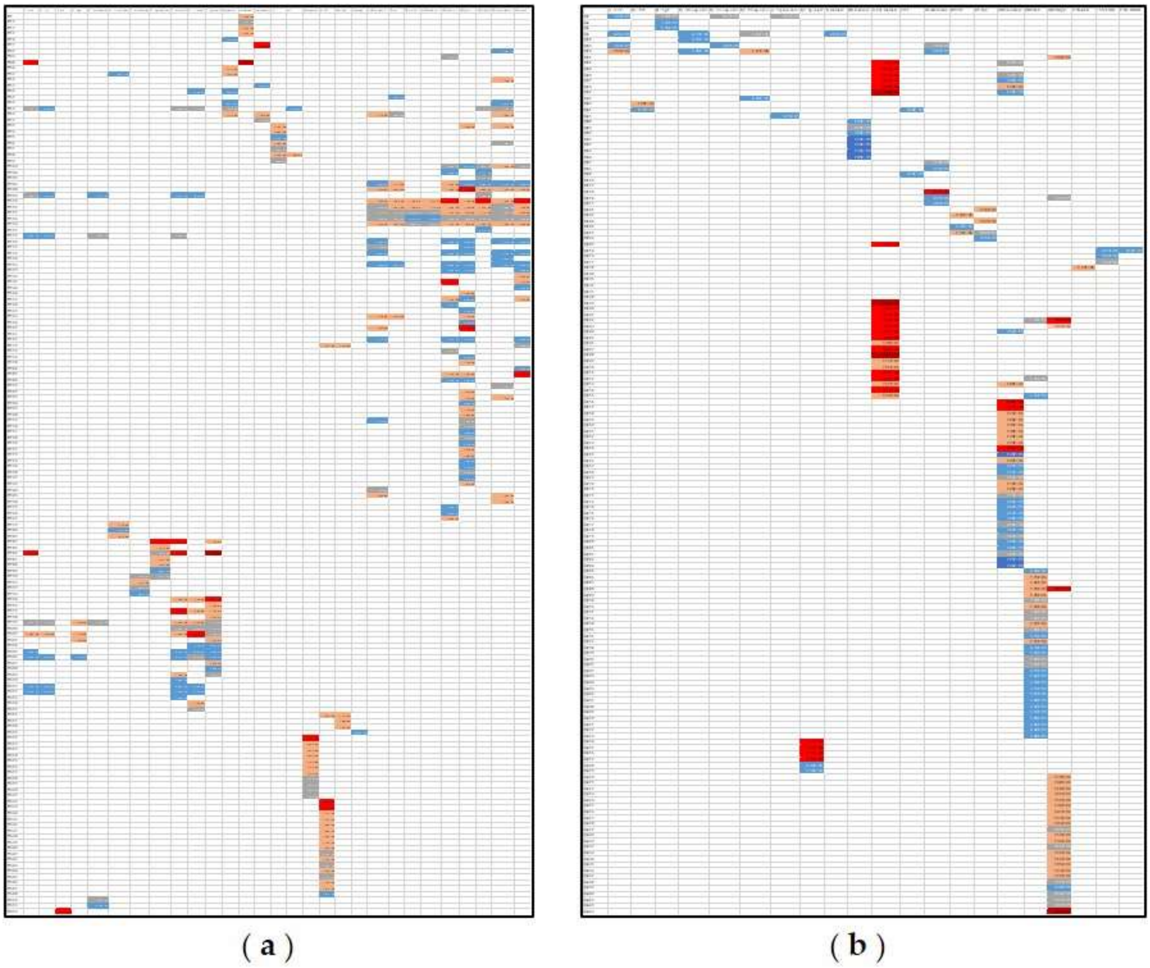

Figure S19. (**a**) Heat map of *P. fallax* HKI727; (**b**) Heat map of *R. jostii* DSM44719. Rows = mass features, columns = producing conditions. Conditions which did not provoke any new mass features are not shown in the heat map. See separate excel file for detailed heat map.

Table S26. Overview of new mass features, proposed compounds, and corresponding producing conditions from selected strains.

ID	Proposed compound	producing conditions
Ba3	cyclo(Tyr-Pro)	Landy medium, LB medium, 40°C, Mg^{2+}-limited GMS medium (GMS MgX)
Ba8	iturin A-4	LB medium
Ba9	iturin A-6	LB medium
Ba10	iturin A-8	LB medium
Ba58	bacillibactin	$Fe3^{+}$-limited GMS medium (GMS FeX)
Cc42	nocardamin	addition of sterile-filtered supernatant of *B. amyloliquefaciens*, *R. jostii* and *S. griseochromogenes*

		(separately)
Pf336	myxochelin A	M9 medium with 1% PO_4^{3-}
Sg117	desmethyl enyl nocardamin	Fe3+-limited GMS medium (GMS FeX)
Sg130	desferrioxamine B + Al	addition of 1% and 3% DMSO

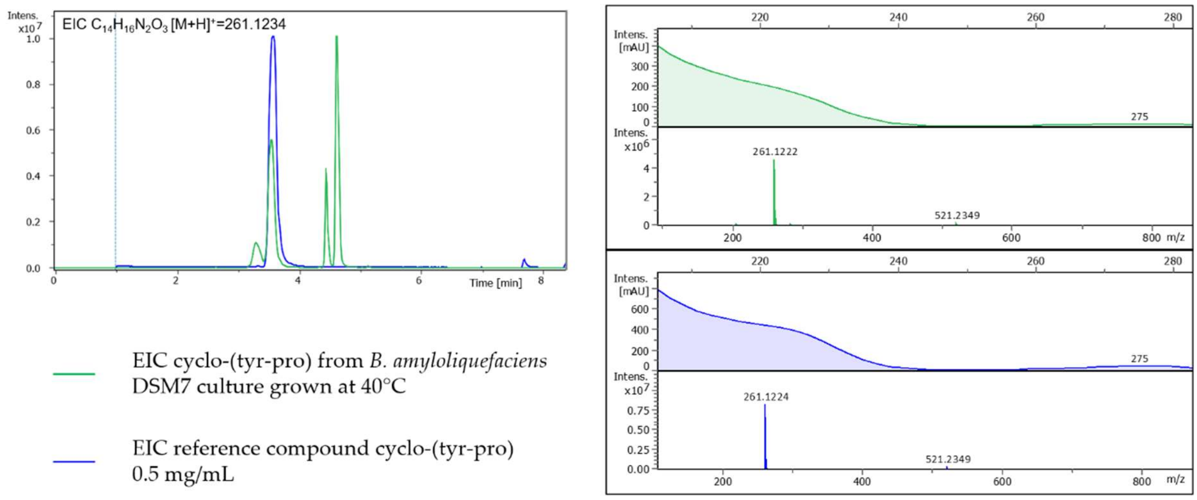

Figure S20. Comparison of Extracted Ion Chromatograms (EICs) and MS spectra of cyclo-(tyr-pro) reference compound (blue) and cyclo-(tyr-pro) in *B. amyloliquefaciens* sample grown at 40°C (green). The UV spectrum is shown above the MS spectrum. Δppm = 0.7.

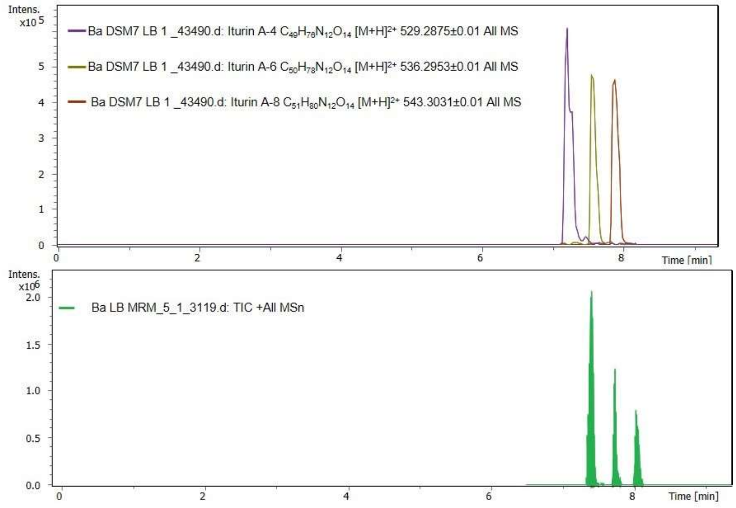

Figure S21. Extracted Ion Chromatograms of putative iturins A4, A-6 and A-8 from *B. amyloliquefaciens* sample grown on LB medium and Total Ion Chromatogram of MS2-experiment from the same sample.

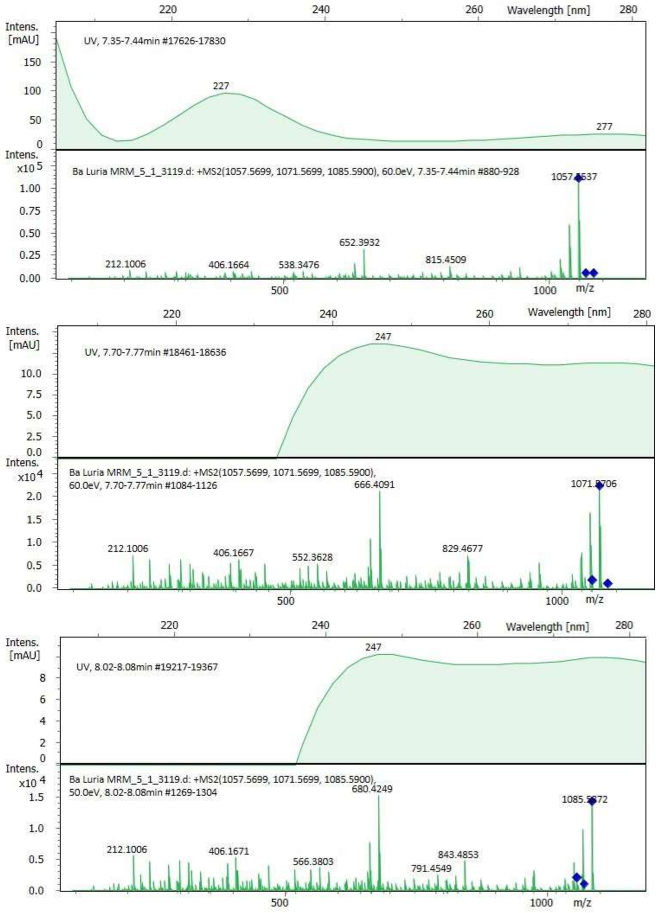

Figure S22. MS2-spectra of putative iturin A-4, A-6 and A-8 masses in *B. amyloliquefaciens* sample grown on LB medium. The UV spectrum is shown above the MS spectrum. Δppm = 12.8 for Iturin A-4, Δppm = 11.5 for Iturin A-6, Δppm = 10.5 for Iturin A-8.

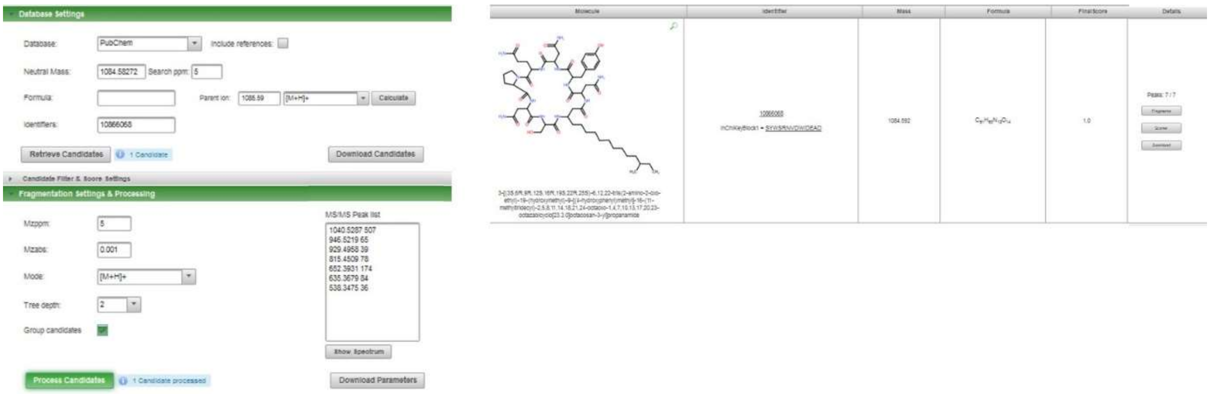

Figure S23. MetFrag settings used for the verification of MS2-fragments of putative iturin A-8 with corresponding PubChem entry #10866068

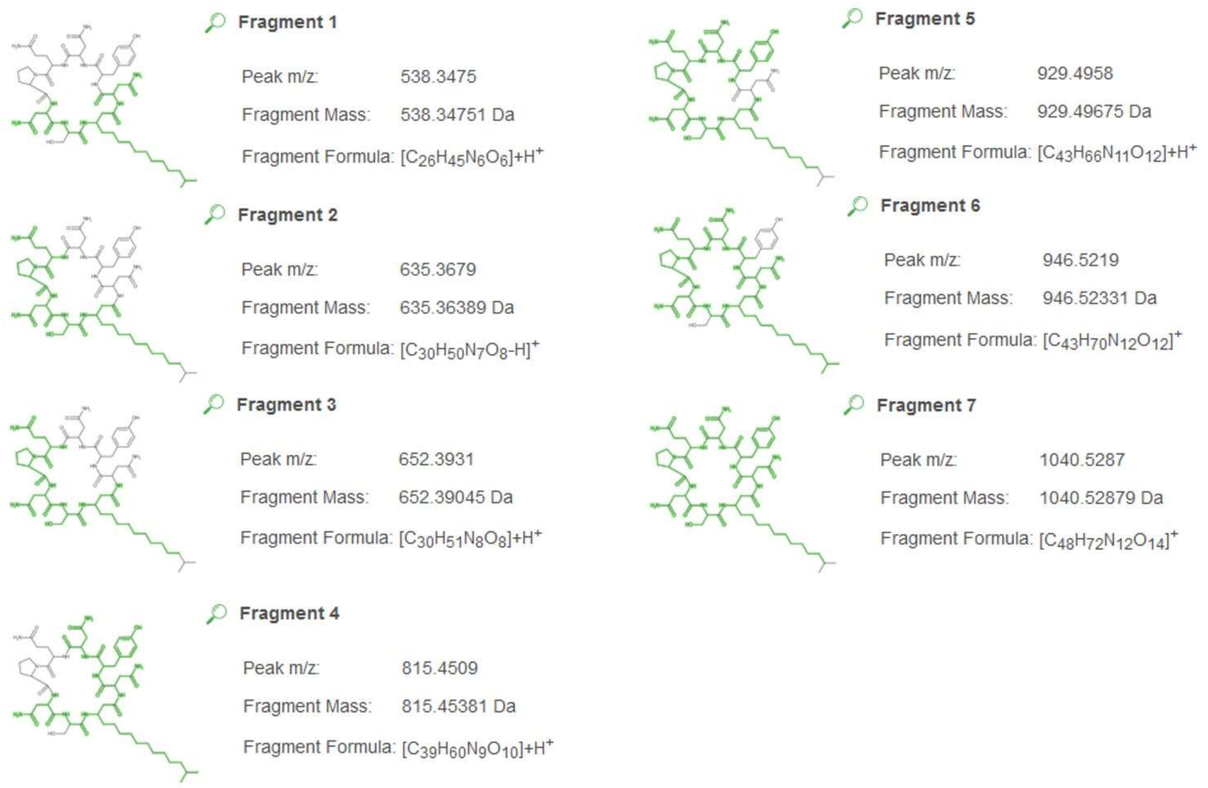

Figure S24. MetFrag explanations of fragments obtained through MS2-analysis of mass 1085.59 (putative iturin A-8) in *B. amyloliquefaciens* DSM7 sample grown on LB medium.

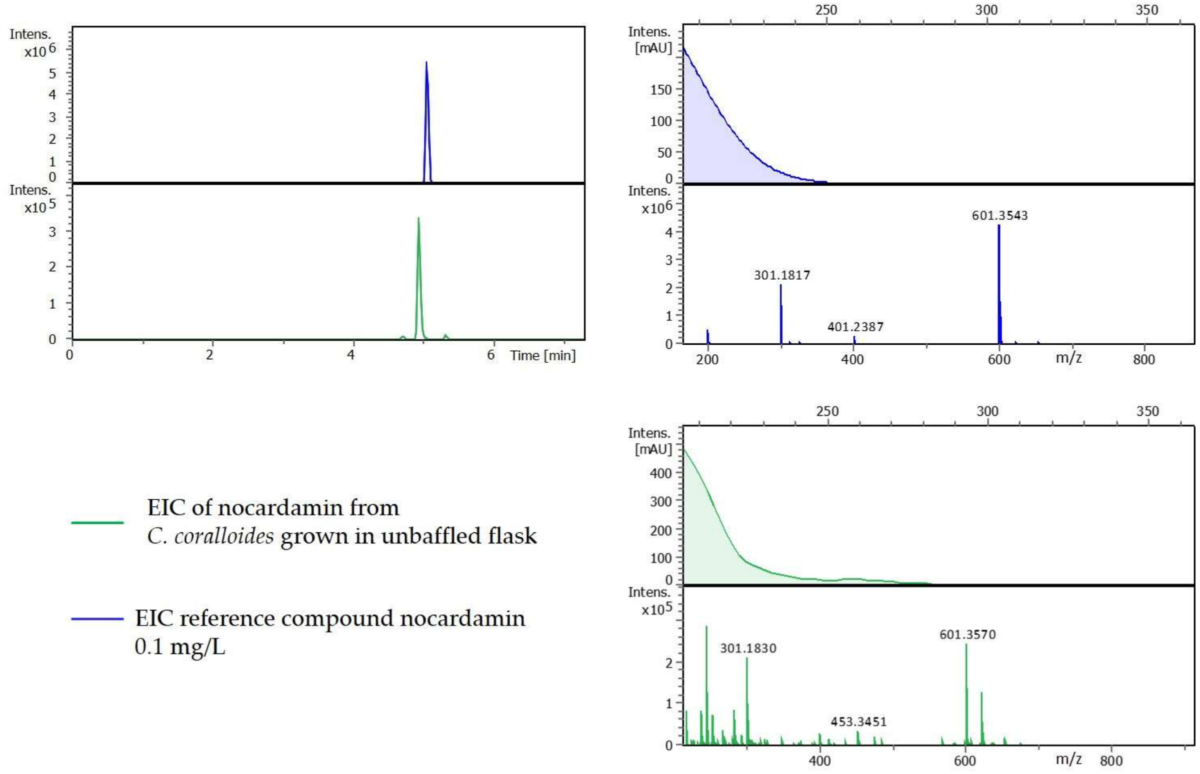

Figure S25. Comparison of Extracted Ion Chromatograms (EICs) and MS spectra of nocardamin reference compound (blue) and nocardamin in *C.coralloides* sample grown in an unbaffled flask (green). The UV spectrum is shown above the MS spectrum. Δppm = 4.4.

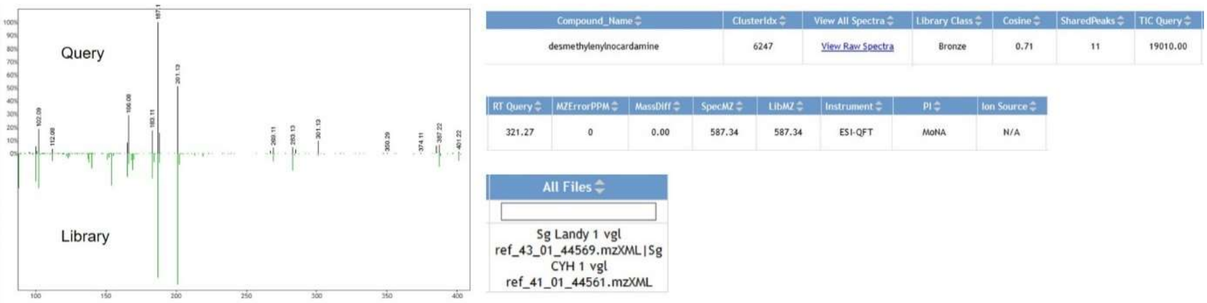

Figure 26. Results of GNPS search of desmethyl enyl nocardamin MS2-spectrum (mirror match and overview). Δppm = 0.

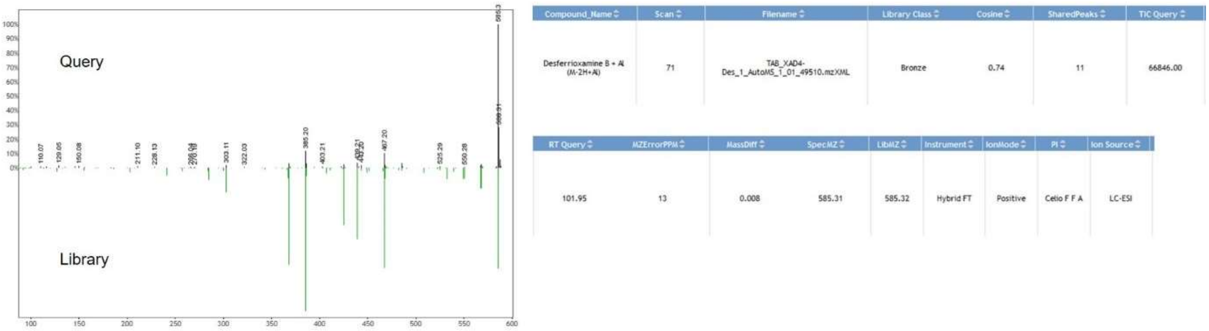

Figure S27. Results of GNPS search of desferrioxamine B + Al MS2-spectrum (mirror match and overview). Δppm = 13.

Table S27. Summary of fragmentation patterns and collision energies (CE) of compounds identified among the new mass features.

Compound and Collision Energy	Fragmentation pattern
cyclo(Tyr-Pro)<br>CE = 35 eV	107.05 1000<br>136.08 501<br>98.06 217<br>113.07 154<br>154.07 246<br>188.11 24
iturin A-4<br>CE = 60 eV	1040.53 517<br>1023.51 301<br>1006.48 159<br>946.52 398<br>929.49 254<br>912.47 162<br>895.45 100<br>846.46 165<br>815.45 387<br>798.43 216<br>781.41 158<br>763.42 177<br>746.40 130<br>718.40 161

	701.38 156<br>684.36 108<br>652.39 1000<br>635.37 611<br>538.35 256<br>406.17 154<br>299.13 311<br>212.10 314
bacillibactin<br>CE = 45 eV	137.02 99<br>159.08 132<br>166.05 89<br>185.09 395<br>194.04 468<br>242.11 102<br>277.08 119<br>295.09 490<br>378.13 1000<br>396.14 237<br>414.15 104<br>453.16 117<br>479.18 235<br>589.18 219<br>672.22 51<br>690.23 44
nocardamin<br>CE = 33 eV	201.1 1000<br>183.11 230<br>168.08 170<br>102.09 170<br>100.03 170<br>283.13 70<br>301.13 40<br>401.24 110
desmethylenyl-nocardamin<br>CE = 28 eV	102.09 190<br>112.06 40<br>166.08 290<br>183.11 180<br>187.1 1000<br>201.13 501<br>269.11 40<br>283.13 50<br>301.13 100<br>387.22 70<br>401.22 10
desferrioxamine B + Al<br>CE = 37.6 eV	585.31 1000 parent ion<br>485.21 32<br>467.20 100

	439.21 33<br>385.19 116<br>368.17 29<br>303.11 16

Table S28. Summary of detected compounds and level of confidence for their identification according to [11].

Compound	Level of Confidence
surfactin	1
myxochelin A	1
desferrioxamine B	1
cyclo(Tyr-Pro)	1
nocardamin	1
bacillibactin	2
nostophycin	2
bacillaene	3
albaflavenone	3
iturin	3

[1] DSMZ GmbH, "Nutrient Medium Composition," available at https://www.dsmz.de/microorganisms/medium/pdf/DSMZ_Medium1.pdf, **2007**.

[2] DSMZ GmbH, "SP Medium Composition," available at https://www.dsmz.de/microorganisms/medium/pdf/DSMZ_Medium222.pdf, **2007**.

[3] DSMZ GmbH, "MD1 Medium Composition," available at https://www.dsmz.de/microorganisms/medium/pdf/DSMZ_Medium1118.pdf, **2008**.

[4] DSMZ GmbH, "TSB Medium Composition," available at https://www.dsmz.de/microorganisms/medium/pdf/DSMZ_Medium545.pdf, **2011**.

[5] DSMZ GmbH, "GYM Medium Composition," available at https://www.dsmz.de/microorganisms/medium/pdf/DSMZ_Medium65.pdf, **2007**.

[6] DSMZ GmbH, "CY/H Medium Composition," available at https://www.dsmz.de/microorganisms/medium/pdf/DSMZ_Medium1542.pdf, **2012**.

[7] DSMZ GmbH, "LB Medium Composition," available at https://www.dsmz.de/microorganisms/medium/pdf/DSMZ_Medium381.pdf, **2007**.

[8] P. Thonart, E. Akpa, B. Wathelet, R. Fuchs, M. Paquot, P. Jacques, H. Budzikiewicz, *Appl. Biochem. Biotechnol.* **2003**, *91–93* (1–9), 551–562. DOI: 10.1385/abab:91-93:1-9:551.

[9] DSMZ GmbH, "M9 Medium Composition," available at https://www.dsmz.de/microorganisms/medium/pdf/DSMZ_Medium1542.pdf, **2007**.

[10] M. Wang, "GNPS Documentation: Mass Spectrometry File Conversion," available at https://ccms-ucsd.github.io/GNPSDocumentation/fileconversion/#conversion-with-msconvert, **n.d.**

[11] A. C. Schrimpe-Rutledge, S. G. Codreanu, S. D. Sherrod, J. A. McLean, *J. Am. Soc. Mass Spectrom.* **2016**, *27* (12), 1897–1905. DOI: 10.1007/s13361-016-1469-y.
